# Chromosomal Radiosensitivity in Sjögren’s Syndrome with and Without Concomitant Malignancy

**DOI:** 10.3390/cells15201855

**Published:** 2026-10-13

**Authors:** Elena Strljic, Lukas Kuhlmann, Jochen Wacker, Hans Geinitz, Stefanie Corradini, Georg Schett, Luitpold V. Distel, Hannah Schenker

**Affiliations:** 1 Department of Radiation Oncology, Friedrich-Alexander-Universität Erlangen-Nürnberg and Universitätsklinikum Erlangen, 91054 Erlangen, Germany; elena.strljic@gmx.de (E.S.); lukas.kuhlmann@uk-erlangen.de (L.K.); stefanie.corradini@uk-erlangen.de (S.C.); 2 Comprehensive Cancer Center Erlangen-Europäische Metropolregion Nürnberg (CCC ER-EMN), 91054 Erlangen, Germany; 3 Department of Internal Medicine 3—Rheumatology and Clinical Immunology, Friedrich-Alexander-Universität Erlangen-Nürnberg and Universitätsklinikum Erlangen, 91054 Erlangen, Germany; jochen.wacker@uk-erlangen.de (J.W.); georg.schett@uk-erlangen.de (G.S.); hannah.schenker@uk-erlangen.de (H.S.); 4 Department of Radiation Oncology, Ordensklinikum Linz Barmherzige Schwestern, 4010 Linz, Austria; hans.geinitz@ordensklinikum.at; 5 Medical Faculty, Johannes Kepler University, 4020 Linz, Austria; 6 Deutsches Zentrum Immuntherapie, Friedrich-Alexander-Universität Erlangen-Nürnberg and Universitätsklinikum Erlangen, 91054 Erlangen, Germany; 7 Department of Internal Medicine 1—Gastroenterology, Pneumology and Endocrinology, Friedrich-Alexander-Universität Erlangen-Nürnberg and Universitätsklinikum Erlangen, 91054 Erlangen, Germany

**Keywords:** Sjögren’s syndrome, systemic lupus erythematosus, individual radiosensitivity, chromosomal aberrations, fluorescence in situ hybridization, breaks per metaphase

## Abstract

**Highlights:**

**What are the main findings?**

- Patients with Sjögren’s syndrome showed increased ex vivo chromosomal radiosensitivity in both non-oncological and oncological cohorts, with no further apparent increase in patients with concomitant malignancy.
- High-grade anti-SSB positivity showed the strongest exploratory association with radiosensitivity and may represent a relevant signal requiring validation in larger cohorts.

**What are the implications of the main findings?**

- The observed pattern may indicate a stronger contribution of disease-associated factors to radiosensitivity in Sjögren’s syndrome than in SLE, where increased radiosensitivity was predominantly associated with concomitant malignancy; however, malignancy-related effects cannot be excluded.
- Prospective studies should integrate ex vivo radiosensitivity with radiotherapy-related toxicity and treatment outcomes to further define its role in individualized risk assessment and radiotherapy planning in Sjögren patients.

**Abstract:**

Connective tissue diseases have long been hypothesized to confer increased radiosensitivity and radiotherapy-related toxicity, although findings vary between disease entities. In a previous institutional study, we found increased radiosensitivity in patients suffering from systemic lupus erythematosus (SLE), primarily in patients with concomitant malignancy. We therefore investigated Sjögren’s syndrome, an autoimmune disease for which evidence has been limited. Ex vivo chromosomal radiosensitivity was assessed in 45 patients with Sjögren’s syndrome (27 non-oncological, 18 oncological) by three-color fluorescence in situ hybridization after 2 Gy irradiation of G0 peripheral blood lymphocytes. Results were compared with 211 healthy controls, 129 breast cancer patients, and the previously characterized SLE cohorts (37 non-oncological, 33 oncological). Demographic, clinical, serological, and treatment-related associations were explored in 31 deeply phenotyped patients. Radiosensitivity was significantly increased in non-oncological Sjögren patients (0.527 breaks per metaphase (B/M)) compared with healthy controls (0.411 B/M; *p* < 0.001) and non-oncological SLE patients (0.404 B/M; *p* = 0.001), and it remained similarly elevated in oncological Sjögren patients (0.527 B/M), whereas in SLE increased radiosensitivity was predominantly associated with concomitant malignancy. High-grade anti-SSB positivity was associated with increased radiosensitivity in the unadjusted analysis (*p* = 0.016), but did not withstand correction for multiple testing (*q* = 0.124). Sjögren’s syndrome is associated with increased ex vivo chromosomal radiosensitivity, with no further apparent increase in patients with concomitant malignancy. This pattern differs from SLE and may suggest a stronger contribution of disease-associated factors. High-grade anti-SSB positivity may represent a relevant serological signal in relation to radiosensitivity, but requires validation in larger cohorts. These findings support closer consideration of individual radiosensitivity in Sjögren’s syndrome and highlight the need for prospective studies integrating ex vivo radiosensitivity with radiotherapy-related toxicity and treatment outcomes in this patient population.

## 1. Introduction

Severe radiotherapy-related toxicity has been reported in patients with Sjögren’s syndrome. In one patient referred to our institution, pronounced skin toxicity with extensive ulceration and prolonged impaired wound healing developed after radiotherapy for cutaneous squamous cell carcinoma, raising the question whether underlying autoimmune disease may contribute to increased radiation susceptibility (This patient was not included in the present study cohort, as referral to our institution occurred after completion of patient inclusion for this study. The case is presented as an illustrative clinical example). Sjögren’s syndrome is a chronic systemic autoimmune disease characterized by lymphocytic infiltration and immune-mediated dysfunction of exocrine glands, mainly affecting the salivary and lacrimal glands. It is driven by abnormal B- and T-cell responses and autoantibody production, including antibodies against Ro/SSA and La/SSB, and clinically ranges from sicca symptoms such as xerostomia and keratoconjunctivitis sicca to fatigue, arthralgia and systemic organ involvement [1,2]. The disease predominantly affects women, with an estimated female-to-male ratio of approximately 10:1 and a prevalence in Germany of around 7 per 10,000 individuals [3]. Importantly, Sjögren’s syndrome is associated with increased mortality, to which malignancies, particularly non-Hodgkin lymphoma, are important contributors [4,5]. In this context, radiotherapy represents an important component of oncological treatment [6,7], but it can still cause acute and late normal-tissue toxicities despite technical advances [8]. This may be particularly relevant in patients with increased individual radiosensitivity. However, evidence specifically addressing radiotherapy-related toxicity in Sjögren’s syndrome is scarce and mainly limited to case reports [9,10], whereas broader studies in connective tissue diseases (CTDs) suggest a heterogeneous but potentially increased risk of radiotherapy-related toxicity in selected CTD subgroups [11,12,13]. In a previous FISH-based study from our institution, we investigated ex vivo chromosomal radiosensitivity in systemic lupus erythematosus (SLE), another systemic autoimmune disease within the spectrum of CTDs. Increased mean radiosensitivity was observed particularly in SLE patients with concomitant malignancy, while radiosensitive individuals were also more frequent in the non-oncological SLE cohort [14]. Given shared autoimmune features between SLE and Sjögren’s syndrome, these findings raised the question whether Sjögren’s syndrome may show a similar pattern of increased radiosensitivity [15]. Individual radiosensitivity is difficult to assess because radiation response involves DNA damage recognition and repair, cell-cycle control, signaling pathways, and cell fate decisions [16]. Functional assays therefore include a variety of approaches, such as clonogenic survival, micronucleus formation, apoptosis, DNA repair, and chromosomal aberration analysis [17]. In contrast to in vivo measurements during radiotherapy, which are influenced by treatment-related factors such as dose distribution and deposited energy, ex vivo irradiation allows a more standardized assessment of individual radiosensitivity [18]. Three-color fluorescence in situ hybridization (FISH)-based analysis of radiation-induced chromosomal aberrations is an established approach for individual radiosensitivity testing, using breaks per metaphase as a suitable parameter for identifying radiosensitive individuals [19]. A key advantage of using peripheral blood lymphocytes in this assay is that they are irradiated in the G0 phase and subsequently stimulated to enter mitosis, thereby avoiding cell-cycle-dependent differences in radiosensitivity [20]. Against this background, we used a three-color FISH assay to investigate ex vivo chromosomal radiosensitivity in patients with Sjögren’s syndrome. We compared non-oncological and oncological Sjögren patients with healthy controls, breast cancer patients, and the previously characterized SLE cohorts from our institution and explored factors associated with increased radiosensitivity.

## 2. Materials and Methods

### 2.1. Study Design and Setting

This observational cohort study was conducted in the Radiation Biology laboratory at the University Hospital of Erlangen in Germany. The study investigated ex vivo radiosensitivity in patients with Sjögren’s syndrome using a three-color fluorescence in situ hybridization (FISH) assay. The study was primarily based on a single-center cohort recruited prospectively in collaboration with the Department of Rheumatology at the same hospital, which served as the clinical recruitment site.

Prospective recruitment took place between September 2022 and May 2023. One additional patient was included in March 2025 under identical study conditions. Additional oncological cases of Sjögren’s syndrome were retrospectively identified from the institutional database of the Institute of Radiation Biology, covering cases submitted between 2016 and 2025 that involved patients with cancer. All patients in our database were referred to us by doctors prior to treatment.

Comparator cohorts obtained from previously established institutional datasets comprised 211 healthy controls, 129 breast cancer patients [21], and previously characterized systemic lupus erythematosus (SLE) cohorts consisting of 37 patients without and 33 patients with malignancy [14]. These cohorts were also evaluated prospectively before the start of treatment.

### 2.2. Study Population

Initially, blood samples from 38 patients with a documented diagnosis of Sjögren’s syndrome were collected during routine outpatient visits at the Department of Rheumatology. Four cases were excluded due to the cytogenetic assay being unsuccessful, primarily due to insufficient lymphocyte stimulation and inadequate metaphase yield. Subsequent chart review of medical records and physician reports revealed alternative diagnoses in three additional cases (sarcoidosis, *n* = 2; Behçet’s disease, *n* = 1), resulting in their exclusion.

**Figure 1:**
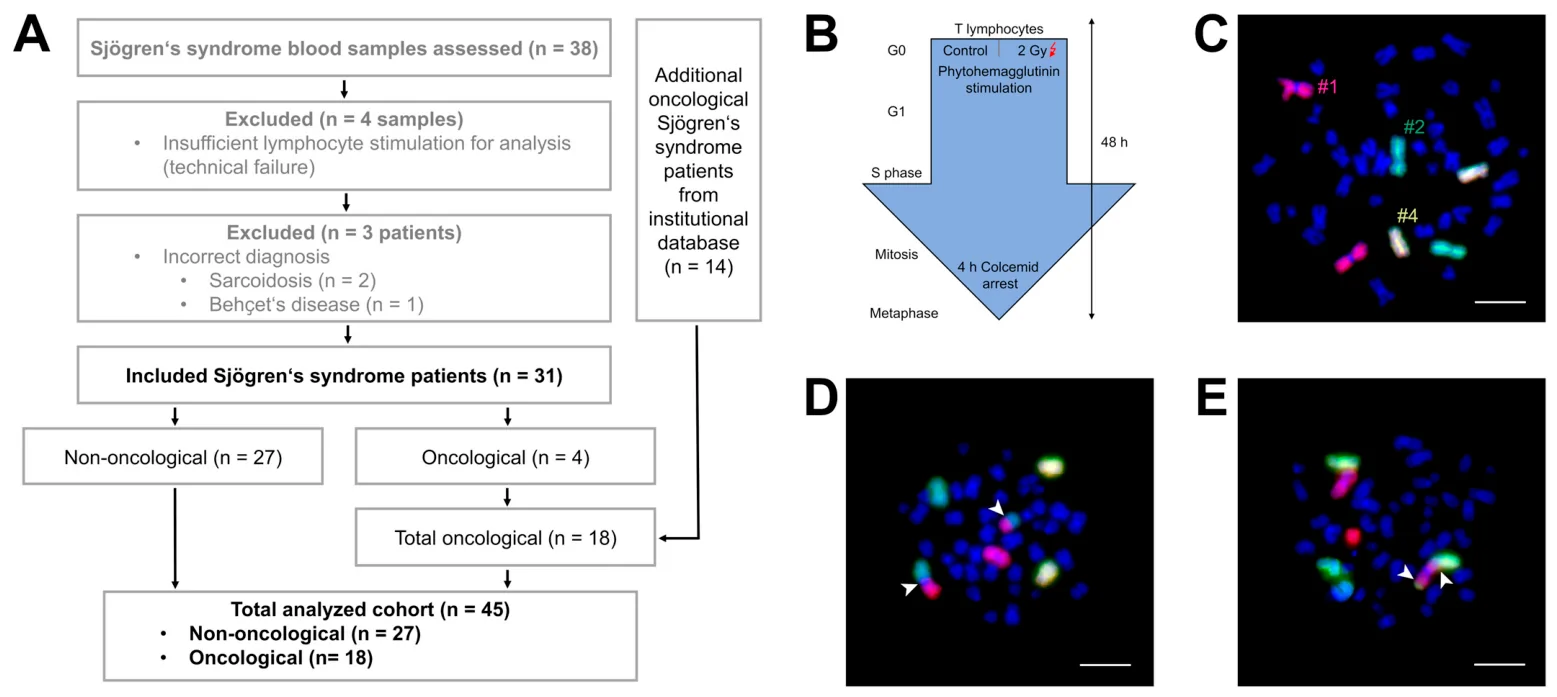
Study cohort assembly and fluorescence in situ hybridization-based analysis of chromosomal aberrations. (**A**) Flow chart illustrating the assembly of the analyzed Sjögren’s syndrome cohort. (**B**) Schematic overview of lymphocyte culture for FISH analysis, including ex vivo irradiation, phytohemagglutinin stimulation, and colcemid-induced metaphase arrest over 48 h. (**C**) Image of a metaphase without visible aberrations showing red-stained chromosome #1, green chromosome #2, and yellow chromosome #4. (**D**) Chromosomes #1 and #2 involved in a translocation, scored as two breaks. (**E**) Chromosomes #1 and #4 involved in an insertion, scored as three breaks. Identifiable break positions are marked by white arrowheads. FISH = fluorescence in situ hybridization. Scale bar = 10 µm.

The final prospectively recruited Sjögren’s syndrome cohort therefore comprised 31 patients. Of these, 27 had no history of malignancy, while four had a concomitant malignant disease. A further 14 patients with Sjögren’s syndrome and malignancy were included from the institutional database. This resulted in a total study cohort of 45 patients, comprising 27 non-oncological and 18 oncological patients with Sjögren’s syndrome (Figure 1A).

### 2.3. Inclusion and Exclusion Criteria

Patients were eligible for inclusion if a diagnosis of Sjögren’s syndrome had been documented by the treating rheumatologist, peripheral blood samples were available, and if the FISH assay could be successfully completed. Patients were excluded if technical assay failure prevented a reliable cytogenetic evaluation or if the initial diagnosis was revised during detailed chart review.

### 2.4. Clinical Data Collection

Clinical and laboratory data were retrospectively extracted from the electronic medical record system of the University Hospital Erlangen, including archived physician reports and laboratory records. Data collection involved demographic and disease-related variables, clinical manifestations, comorbidities, serological and laboratory parameters, and treatment history.

Demographic parameters included age, sex, body weight, height, and body mass index. Disease-related variables comprised age at Sjögren’s syndrome diagnosis and disease duration at the time of blood sampling. Clinical manifestations covered EULAR Sjögren’s Syndrome Disease Activity Index (ESSDAI)-relevant organ involvement and systemic features, as well as other common symptoms associated with Sjögren’s syndrome, such as sicca syndrome, Raynaud’s phenomenon, joint involvement and gastrointestinal involvement. Comorbidities included the presence of malignancy (used for subgroup classification), as well as rheumatological and other frequently observed non-rheumatological comorbidities. Laboratory parameters comprised autoantibody profiles, including ANA and Sjögren-specific antibodies (anti-SSA, anti-SSB, anti-Ro52), as well as hematological abnormalities and immunological markers such as complement levels, immunoglobulin levels, serum protein electrophoresis, immunofixation and free light chains. Anti-SSA, anti-SSB, and anti-Ro52 results were obtained from routine clinical laboratory testing performed at University Hospital Erlangen as part of rheumatological care. Autoantibodies were assessed using a line immunoblot assay (EUROLINE ANA Profil et Mi-2, Ku, DFS70 (IgG), EUROIMMUN Medizinische Labordiagnostika AG, Lübeck, Germany). Test strips were scanned using a flatbed scanner and evaluated with EUROLineScan software (V3.4.37). According to the manufacturer’s interpretation criteria for flatbed-scanner evaluation, a signal intensity >50 was classified as strongly positive (+++); in the present study; this category was termed high-grade positivity. Treatment data included current and previous immunosuppressive therapies. For glucocorticoids, both exposure and dose were recorded due to their potential influence on lymphocyte stimulation in the cytogenetic assay, whereas other immunomodulatory and immunosuppressive therapies were primarily analyzed as binary exposure variables.

Comprehensive clinical data were available for all prospectively recruited patients with Sjögren’s syndrome, including the four oncological cases within this cohort. Additional oncological cases retrieved from the institutional database provided only limited clinical information restricted to demographic data, malignancy status, and rheumatological comorbidity, alongside radiosensitivity measurements.

### 2.5. Three-Color Fluorescence In Situ Hybridization Assay

Peripheral venous blood samples were obtained from all study participants. For each patient, 5 mL whole blood was exposed ex vivo to 2 Gy ionizing radiation using a 6 MV linear accelerator (Varian, Palo Alto, CA, USA) to induce chromosomal aberrations. Irradiation was performed in a 62 mm thick acrylic block that contained a 9 mm radius hole at a depth of 20 mm. To account for the buildup effect of ionizing radiation, there were 11 mm of acrylic in front of the 8.5 mm radius blood tube. The entire acrylic block, with a surface area of 100 mm x 140 mm, was within the radiation field. Dosimetry is verified as part of regular, daily checkups. Irradiation was performed at a dose rate of 1.0 Gy per minute at room temperature. A second 5 mL aliquot served as a non-irradiated control for assessment of background chromosomal aberrations.

The peripheral blood lymphocytes were irradiated in the G0 phase and then cultured in an RPMI-based medium containing phytohemagglutinin (PHA-L Pure, Biochrom, Berlin, Germany) to stimulate cell cycling and mitotic entry. After 44 h of incubation at 37 °C, colcemid was added for four hours to arrest the dividing cells in metaphase. Chromosome preparation was then performed according to the institutional standard protocol (Figure 1B).

Three-color fluorescence in situ hybridization (FISH) was performed using probes targeting chromosomes 1, 2 and 4. The chromosomes were stained red, green, and yellow using the fluorophores FITC, rhodamine, and a combination of both. Nuclear DNA was counterstained with DAPI.

### 2.6. Image Acquisition and Analysis

Metaphases within the hybridized area were identified automatically by a fluorescence microscope (Zeiss Axioplan 2, Göttingen, Germany) using Metafer 4 imaging software (V3.10.7, MetaSystems, Altlussheim, Germany). Automated screening was performed with 100 times magnification and individual metaphase images were acquired at 630 magnification (Figure 1C–E). The digital images were then analyzed using semi-automated image analysis software (Biomas, Lab of Radiation Biology, Erlangen, Germany). On average, 110 (range 92–134) images were analyzed for the background value and 191 (range 57–230) images for the 2 Gy value. Images were analyzed only if all six stained chromosomes were present. The patients have been pseudonymized and are therefore anonymous to those analyzing the data.

The breakage rate was determined for each metaphase based on the minimum number of DNA double-strand breaks required to explain the observed chromosomal rearrangements. Accordingly, fragments were scored as one break; translocations, dicentrics, and rings as two breaks; and complex aberrations as the estimated minimum number of breaks underlying the respective structure.

Background aberrations detected in the non-irradiated control sample were subtracted from radiation-induced breaks after 2 Gy exposure. Individual radiosensitivity was expressed as the background-corrected breaks per metaphase (B/M) value after 2 Gy irradiation; mean radiosensitivity refers to the average of these individual B/M values within a given cohort or subgroup. B/M values ≥0.55 were considered indicative of increased radiosensitivity. The cutoff value was derived from the B/M values of healthy individuals. A normal distribution was fitted and the mean plus three standard deviations was chosen as the threshold for increased radiation sensitivity. Throughout the entire study period, identical materials and equipment were used, and the essential steps of the protocols and the analysis were carried out in exactly the same way.

### 2.7. Statistics and Figure Generation

Statistical analyses were performed using GraphPad Prism (version 10.6.0, GraphPad Software, La Jolla, CA, USA). Figures were generated using GraphPad Prism and assembled into composite panels using GIMP (3.0, 2025, GIMP Development Team). Normality was assessed using the Shapiro–Wilk test and Q-Q plots. Parametric tests were applied where data were considered approximately normally distributed based on normality testing, with additional consideration of sample size for larger groups.

Comparisons between two independent groups were performed using unpaired *t*-tests with Welch’s correction for normally distributed data and Mann–Whitney U tests for non-normally distributed data or smaller subgroup sizes. Comparisons across more than two groups were conducted using the Kruskal–Wallis test due to small subgroup sizes and the non-parametric data structure. Categorical variables were analyzed using Fisher’s exact test. Correlation analyses were assessed using simple linear regression. Statistical testing was only performed when subgroup sizes were sufficient (*n* = 3 per group).

All statistical tests were two-sided, and a *p*-value < 0.05 was considered statistically significant. Primary cohort comparisons were predefined and hypothesis-driven. To account for potential confounding by age and sex in the primary cohort comparisons, multiple linear regression analyses were performed, with background-corrected B/M after 2 Gy irradiation as the dependent variable and cohort, age, and sex as independent variables. Analyses of demographic, disease-related, clinical, serological, laboratory, and treatment-related factors within the deeply phenotyped Sjögren subcohort (*n* = 31) were considered exploratory. Given the number of inferential tests performed within the serological and laboratory analyses, *p* values from all inferential tests in these analyses were additionally adjusted using the Benjamini–Hochberg false discovery rate procedure with an FDR of 5%. Adjusted *p* values are reported as q values.

AI-assisted language tools, such as DeepL, were used for linguistic and stylistic refinement; all scientific content, analyses, and interpretations were developed and verified by the authors.

### 2.8. Ethics Approval and Consent to Participate

Written informed consent for the scientific use of biological material and clinical data was obtained from all patients and healthy controls. The study, including the use of individual patient data and retrospective analysis of medical records, was approved by the Ethics Committee of the University Hospital Erlangen (ethics approval: 21_19 B and 25-367-B).

## 3. Results

### 3.1. Cohort Characteristics

We were interested in the individual radiosensitivity of patients with Sjögren’s syndrome. In total, 455 individuals were analyzed, comprising 211 healthy controls, 129 breast cancer patients, 37 non-oncological SLE patients, 33 oncological SLE patients, 27 non-oncological Sjögren patients, and 18 oncological Sjögren patients.

Female predominance was observed across all disease cohorts, being most pronounced in breast cancer and oncological Sjögren patients (both 100%). The respective proportions were 57.8% in healthy individuals, 75.7% in non-oncological SLE, 81.8% in oncological SLE, and 92.6% in non-oncological Sjögren patients. Mean age was 50.8 years (range 19–81) in healthy controls, 57.3 years (range 23–91) in breast cancer patients, 49.6 years (range 21–77) in non-oncological SLE, 56.6 years (range 44–80) in oncological SLE, 48.1 years (range 19–74) in non-oncological Sjögren patients, and 58.6 years (range 24–78) in oncological Sjögren patients.

Detailed clinical, serological, and treatment-related data used for the subsequent analyses were available only for a deeply phenotyped subcohort of patients with Sjögren’s syndrome (*n* = 31). This subcohort included all non-oncological Sjögren’s syndrome patients and four prospectively recruited oncological Sjögren’s syndrome patients. Listed demographic, clinical, therapeutic, and laboratory baseline characteristics are provided in Supplementary Tables S1–S3.

### 3.2. Radiosensitivity in Sjögren’s Syndrome Compared with Healthy Controls, Breast Cancer, and SLE Cohorts

**Figure 2:**
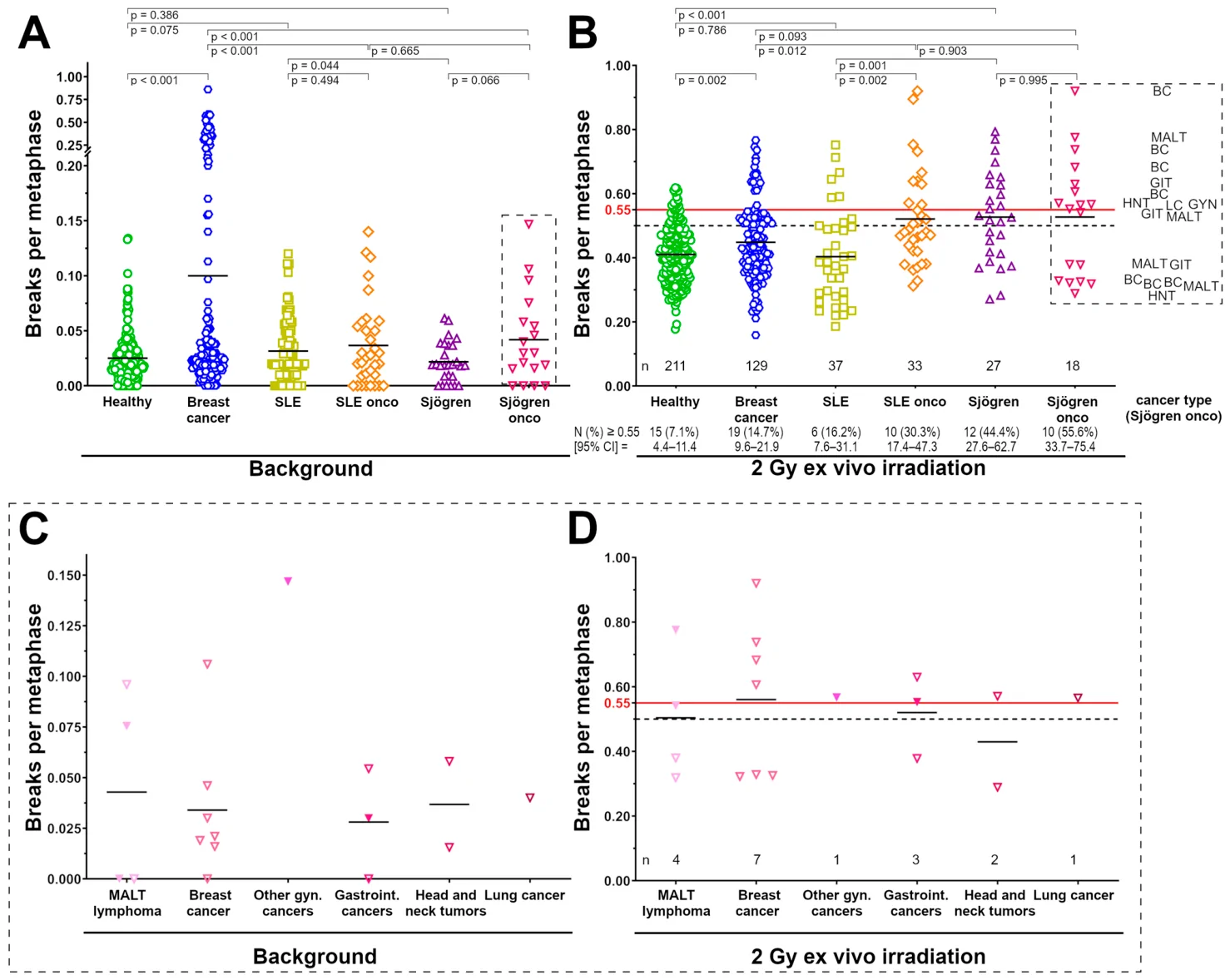
Chromosomal aberrations in the Sjögren cohort and control groups scored as breaks per metaphase. (**A**) Background values in 211 healthy controls, 129 breast cancer patients, 37 non-oncological and 33 oncological SLE patients, and 27 non-oncological and 18 oncological Sjögren’s syndrome patients. (**B**) Background-corrected breaks per metaphase after ex vivo irradiation with 2 Gy ionizing radiation in the same cohorts indicate radiosensitivity. Associated cancer types are indicated for the oncological Sjögren cohort. (**C**) Background values within the oncological Sjögren cohort stratified by cancer subtype. (**D**) Background-corrected breaks per metaphase after 2 Gy irradiation within the oncological Sjögren cohort stratified by cancer subtype. Black horizontal lines indicate mean values. Brackets indicate statistical comparisons with corresponding *p* values. Red horizontal lines indicate the cutoff for increased radiosensitivity at ≥0.55 breaks per metaphase; dashed black horizontal lines are set at 0.5 B/M. In (**B**), the number and percentage of individuals with a B/M ≥ 0.55, together with the corresponding 95% CI, are shown below the x-axis. Filled symbols in (**C**,**D**) indicate prospectively recruited oncological Sjögren cases. SLE = systemic lupus erythematosus; onco = oncological cohort; MALT = mucosa-associated lymphoid tissue lymphoma; BC = breast cancer; GYN = other gynecological cancers; GIT = gastrointestinal tumor; HNT = head and neck tumor; LC = lung cancer. Statistical analyses were performed using unpaired *t*-tests with Welch’s correction for (**A**,**B**) and Kruskal–Wallis tests for (**C**,**D**).

We first assessed background rates of chromosomal aberrations (0 Gy), reflecting baseline genomic instability potentially related to environmental exposures or impaired DNA damage response (Figure 2A). Mean background values were 0.025 ± 0.021 B/M in healthy controls, 0.100 ± 0.158 B/M in breast cancer patients, 0.032 ± 0.029 B/M in non-oncological SLE patients, 0.037 ± 0.038 B/M in oncological SLE patients, 0.022 ± 0.018 B/M in non-oncological Sjögren patients, and 0.042 ± 0.040 B/M in oncological Sjögren patients. No significant differences were observed between healthy controls and either non-oncological autoimmune cohort, namely non-oncological Sjögren’s syndrome (*p* = 0.386) and SLE patients (*p* = 0.075). In contrast, breast cancer patients showed significantly elevated background damage compared with healthy controls (*p* < 0.001). Within disease entities, background levels did not differ between non-oncological and oncological cohorts in either Sjögren’s syndrome (*p* = 0.066) or SLE (*p* = 0.494), suggesting no major influence of malignancy on baseline chromosomal instability. Direct comparison of both autoimmune diseases revealed slightly lower background values in non-oncological Sjögren patients compared with non-oncological SLE patients (*p* = 0.044), whereas the oncological cohorts had comparable values (*p* = 0.665).

Next, we examined radiosensitivity after ex vivo irradiation with 2 Gy (Figure 2B). Mean background-corrected B/M values were 0.411 ± 0.088 in healthy controls, 0.449 ± 0.119 in breast cancer patients, 0.404 ± 0.148 in non-oncological SLE patients, 0.521 ± 0.146 in oncological SLE patients, 0.527 ± 0.139 in non-oncological Sjögren patients, and 0.527 ± 0.178 in oncological Sjögren patients. While background-corrected radiosensitivity values of non-oncological SLE patients and healthy controls remained comparable to each other (*p* = 0.786), radiosensitivity in the non-oncological Sjögren cohort was markedly increased compared with healthy controls (*p* < 0.001). Breast cancer patients likewise showed significantly higher radiosensitivity than healthy controls (*p* = 0.002). Direct comparison between autoimmune diseases confirmed significantly higher radiosensitivity in non-oncological Sjögren patients compared with non-oncological SLE patients (*p* = 0.001), while radiosensitivity levels in the corresponding oncological cohorts were comparable (*p* = 0.903), clustering at similarly elevated levels. Assessing the effect of malignancy within each disease, radiosensitivity did not differ between non-oncological and oncological Sjögren cohorts (*p* = 0.995), indicating that concomitant malignancy did not appear to further increase radiosensitivity in Sjögren’s syndrome. In contrast, oncological SLE patients demonstrated significantly higher radiosensitivity than their non-oncological counterparts (*p* = 0.002), highlighting a strong effect of malignancy in SLE. Notably, oncological SLE patients even exceeded breast cancer patients in radiosensitivity (*p* = 0.012), while a similar trend in oncological Sjögren patients did not reach statistical significance (*p* = 0.093). Descriptive statistics (mean ± SD), mean differences, and corresponding 95% confidence intervals for the unadjusted primary cohort comparisons at 0 Gy and after 2 Gy irradiation are provided in Supplementary Table S4.

To account for potential confounding by age and sex, the primary cohort comparisons were additionally assessed using multiple linear regression (Table 1). Adjustment for age and sex largely confirmed the unadjusted findings. Of note, the comparison between oncological Sjögren and breast cancer patients reached statistical significance after adjustment (*p* = 0.008).

Overall, the pattern observed in Sjögren’s syndrome may point toward a greater contribution of disease-associated factors compared with SLE, in which increased radiosensitivity was primarily associated with the presence of concomitant malignancy.

### 3.3. Influence of Cancer Subtype Within the Oncological Sjögren Cohort

In order to assess whether malignancy subtype influenced radiosensitivity, the oncological Sjögren cohort was stratified by tumor entity (Figure 2C,D; Table 2). The cohort comprised MALT lymphoma (*n* = 4), breast cancer (*n* = 7), gastrointestinal tumors (*n* = 3), head and neck tumors (*n* = 2), lung cancer (*n* = 1), and other gynecological malignancies (*n* = 1). Given the small subgroup sizes, particularly for entities with *n* ≤ 2, meaningful subgroup-level interpretation is limited. Accordingly, statistical comparisons were restricted to tumor entities with *n* ≥ 3 (MALT lymphoma, breast cancer, gastrointestinal tumors). Overall, no significant differences between evaluable tumor subgroups were detected in baseline chromosomal breakage (Kruskal–Wallis *p* = 0.987) or after 2 Gy irradiation (*p* = 0.924). Within the limitations of the small subgroup sizes, these exploratory analyses did not indicate that the elevated radiosensitivity observed in Sjögren’s syndrome was driven by a specific cancer subtype.

### 3.4. Influence of Demographic and Disease-Related Factors on Radiosensitivity in Sjögren’s Syndrome

To explore potential determinants of radiosensitivity, we next investigated whether demographic and disease-related factors, including sex, age, and disease duration, influence chromosomal instability and radiosensitivity in patients with Sjögren’s syndrome.

**Figure 3:**
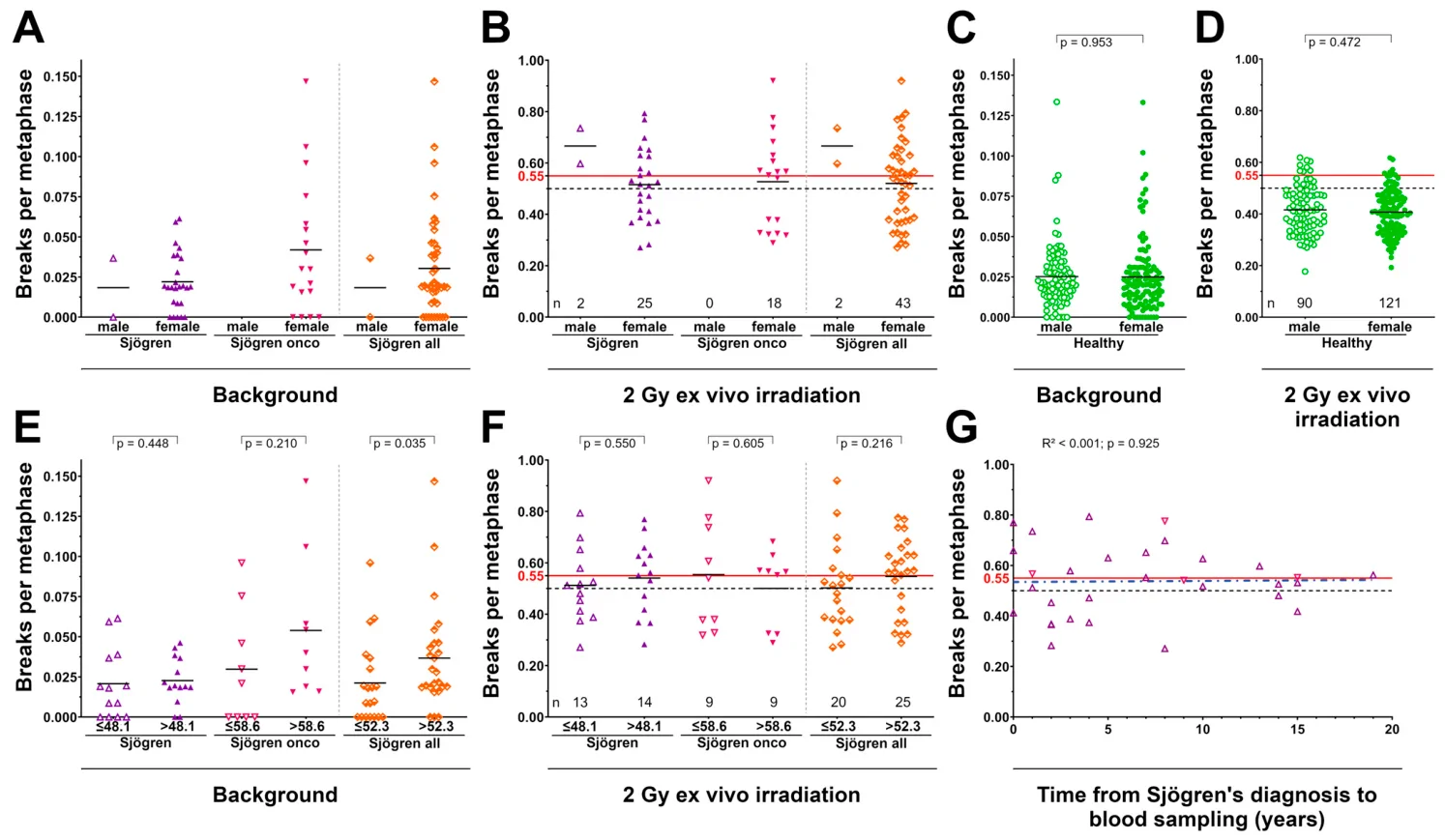
Association of sex, age, and disease duration with chromosomal aberrations in Sjögren’s syndrome. (**A**) Background values in 27 non-oncological and 18 oncological Sjögren’s syndrome patients, as well as in the overall Sjögren cohort (*n* = 45), separated by sex. (**B**) Background-corrected breaks per metaphase after 2 Gy irradiation indicate radiosensitivity according to the same stratification. (**C**) Background values in 211 healthy individuals separated by sex. (**D**) Background-corrected breaks per metaphase after 2 Gy irradiation indicate radiosensitivity in healthy individuals separated by sex. (**E**) Background values in non-oncological, oncological, and overall Sjögren’s syndrome cohorts stratified according to the respective mean age in years. (**F**) Background-corrected breaks per metaphase after 2 Gy irradiation indicate radiosensitivity according to the same stratification. (**G**) Association between disease duration and radiosensitivity after 2 Gy irradiation in the deeply phenotyped Sjögren cohort (*n* = 31). Black horizontal lines indicate mean values. Brackets indicate statistical comparisons with corresponding *p* values. Red horizontal lines indicate the cutoff for increased radiosensitivity at ≥0.55 breaks per metaphase; dashed black horizontal lines are set at 0.5 B/M. onco = oncological cohort; all = overall cohort. In (**G**), purple upward triangles indicate non-oncological Sjögren patients and pink downward triangles indicate oncological Sjögren patients; the dashed blue line indicates the linear regression line. Statistical analyses were performed using unpaired *t*-tests with Welch’s correction for (**C**,**D**), Mann–Whitney U tests for (**E**,**F**), and simple linear regression for (**G**).

Sex-stratified analyses of the Sjögren cohorts revealed no robust or interpretable differences in background chromosomal aberrations (0 Gy) or radiosensitivity after 2 Gy irradiation (Figure 3A,B). Visual inspection suggested slightly higher background values in female patients compared with males when considering the overall Sjögren cohort, whereas the opposite trend was observed after irradiation, with higher radiosensitivity levels in male patients. However, these observations are limited by the highly unbalanced sex distribution, with only two male patients in the entire Sjögren cohort and no males in the oncological subgroup. As a result, no formal statistical comparisons were performed. To further assess potential sex effects, we analyzed the healthy control cohort (*n* = 211), which showed a more balanced sex distribution (Figure 3C,D). In this cohort, neither background chromosomal aberrations (*p* = 0.953) nor radiosensitivity after 2 Gy irradiation (*p* = 0.472) differed between male and female individuals, indicating that sex does not significantly influence radiosensitivity in healthy subjects.

We next examined the impact of age by stratifying the Sjögren cohorts according to the respective mean age within each subgroup. For background chromosomal aberrations (Figure 3E), higher values were observed in older patients compared to younger individuals when considering the overall Sjögren cohort, reaching statistical significance (*p* = 0.035). Any apparent differences in the non-oncological and oncological subgroups did not reach statistical significance (non-oncological: *p* = 0.448; oncological: *p* = 0.210). After 2 Gy irradiation (Figure 3F), no significant age-dependent differences in radiosensitivity were detected in any subgroup (overall: *p* = 0.216; non-oncological: *p* = 0.550; oncological: *p* = 0.605).

Finally, we assessed the impact of disease duration on radiosensitivity by analyzing the association between radiosensitivity after 2 Gy irradiation and the time interval between Sjögren’s syndrome diagnosis and blood sampling (Figure 3G). Linear regression analysis revealed no significant correlation (R^2^ < 0.001, *p* = 0.925).

Therefore, age and disease duration do not appear to substantially influence radiosensitivity in patients with Sjögren’s syndrome, while no clear effect of sex could be determined.

### 3.5. Clinical Features and Their Association with Radiosensitivity in Sjögren’s Syndrome

A subsequent analysis addressed the association between clinical characteristics and radiosensitivity in patients with Sjögren’s syndrome, focusing on disease activity, symptoms, and comorbidities within the deeply phenotyped subcohort.

**Figure 4:**
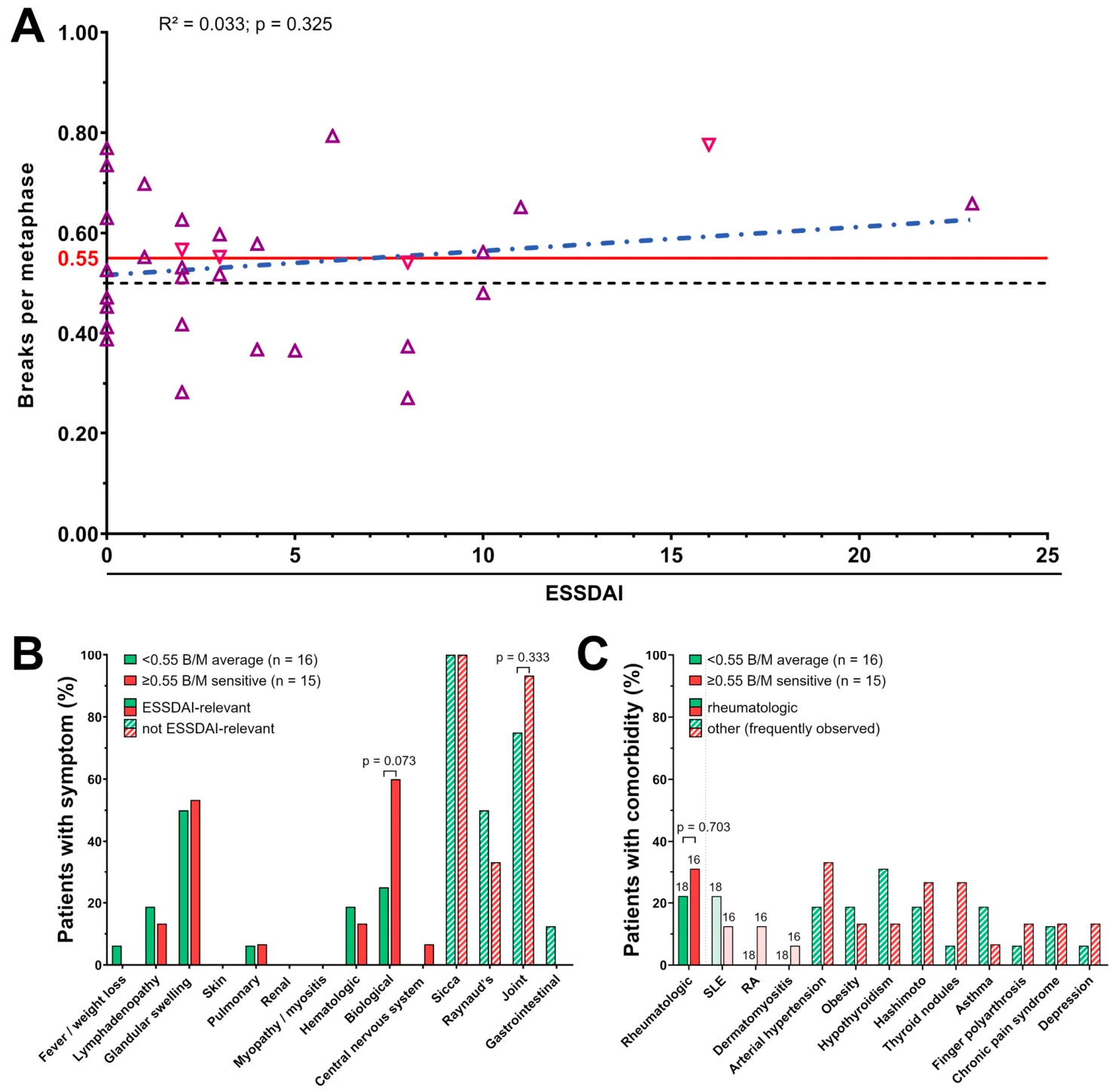
Association of ESSDAI score, symptoms, and comorbidities with radiosensitivity in the deeply phenotyped Sjögren cohort. (**A**) Association between ESSDAI score and radiosensitivity after 2 Gy irradiation. The red horizontal line indicates the cutoff for increased radiosensitivity at ≥0.55 breaks per metaphase; the dashed black horizontal line is set at 0.5 B/M. Purple upward triangles indicate non-oncological Sjögren patients and pink downward triangles indicate oncological Sjögren patients; the dashed blue line indicates the linear regression line. (**B**) Distribution of symptoms in non-radiosensitive <0.55 B/M (green bars) and radiosensitive ≥0.55 B/M (red bars) Sjögren patients. (**C**) Distribution of comorbidities in non-radiosensitive and radiosensitive Sjögren patients. Numbers above rheumatological comorbidity bars indicate the respective subgroup sizes due to additionally available comorbidity data. Brackets in (**B**,**C**) indicate statistical comparisons with corresponding *p* values. ESSDAI = EULAR Sjögren’s Syndrome Disease Activity Index; B/M = breaks per metaphase; SLE = systemic lupus erythematosus; RA = rheumatoid arthritis. Statistical analyses were performed using simple linear regression for (**A**) and Fisher’s exact tests for (**B**,**C**).

Regarding disease activity, as measured by the ESSDAI score [22], a weak positive trend toward increased chromosomal aberrations after 2 Gy irradiation was observed (Figure 4A). However, linear regression analysis did not demonstrate a significant association (R^2^ = 0.033, *p* = 0.325).

We then assessed the distribution of clinical symptoms between radiosensitive and non-radiosensitive patients (Figure 4B). While most symptoms were similarly distributed between groups, some features showed a tendency toward higher prevalence in radiosensitive individuals. In particular, biological and joint involvement were more frequent in radiosensitive individuals, although these differences did not reach statistical significance (biological: *p* = 0.073; joint: *p* = 0.333).

Similarly, the prevalence of comorbidities showed no meaningful association with radiosensitivity (Figure 4C). While rheumatological comorbidities, when considered as a composite category, were slightly more frequent among radiosensitive patients, this observation was not statistically significant (*p* = 0.703).

Overall, we did not find a significant relationship between increased radiosensitivity and disease activity, symptom profiles, or comorbidities.

### 3.6. Autoantibody Profiles and Additional Serological Features in Relation to Radiosensitivity

We further investigated why some patients with Sjögren’s syndrome are more sensitive to ionizing radiation and others are not. Thus, we aimed to identify whether a certain immunological signature was related to the increased B/M levels after 2 Gy irradiation by analyzing autoantibody profiles and additional serological parameters within the deeply phenotyped Sjögren subcohort (*n* = 31).

**Figure 5:**
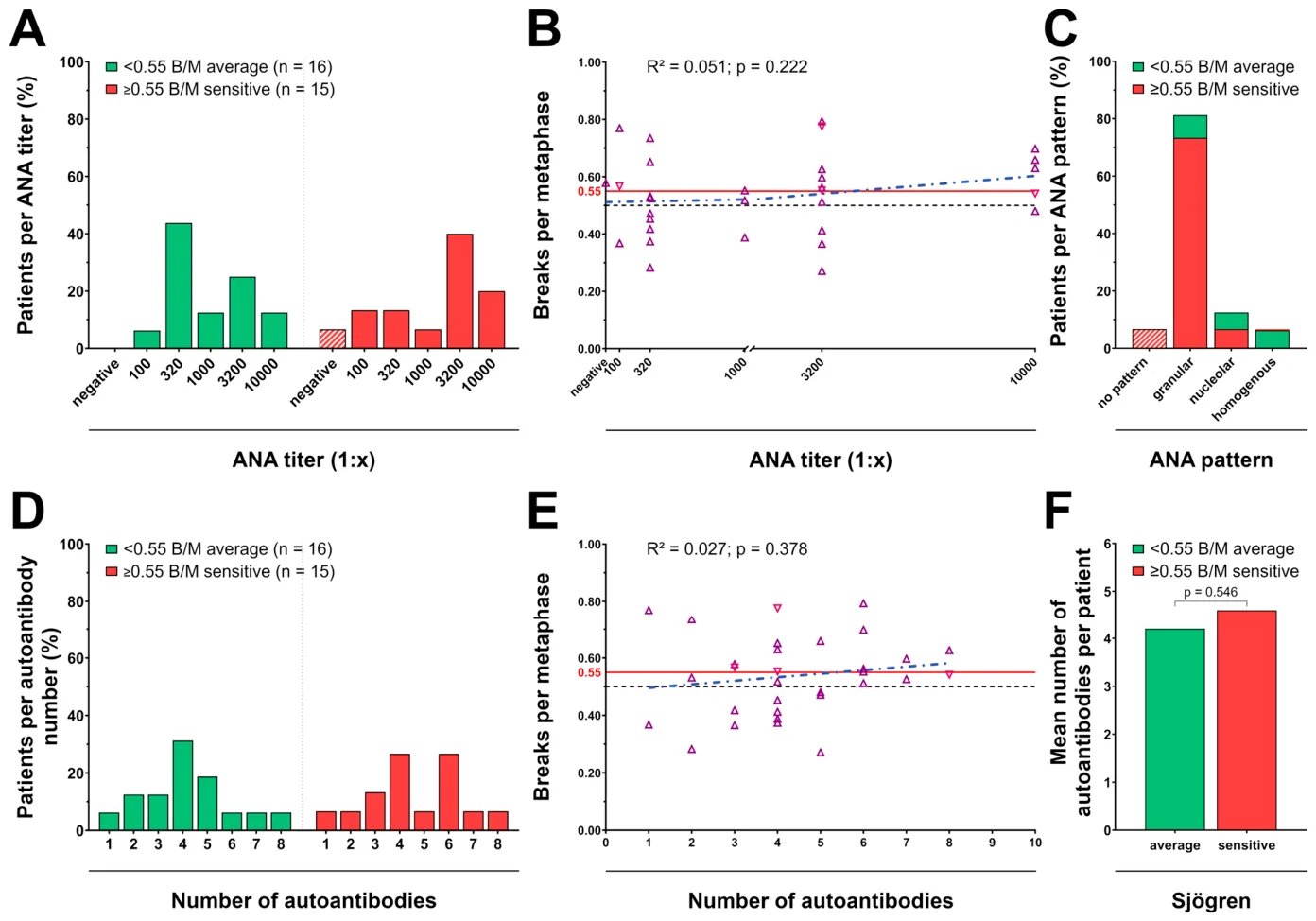
Association of ANA characteristics and autoantibody burden with radiosensitivity in the deeply phenotyped Sjögren cohort. (**A**) Distribution of ANA titers in non-radiosensitive <0.55 B/M (green bars) and radiosensitive ≥0.55 B/M (red bars) Sjögren patients. (**B**) Association between ANA titer and radiosensitivity after 2 Gy irradiation. (**C**) Distribution of ANA staining patterns in non-radiosensitive and radiosensitive Sjögren patients. No pattern indicates ANA-negative samples. (**D**) Distribution of the number of autoantibodies per patient in non-radiosensitive and radiosensitive Sjögren patients. (**E**) Association between the number of autoantibodies per patient and radiosensitivity after 2 Gy irradiation. (**F**) Mean number of autoantibodies per patient in non-radiosensitive and radiosensitive Sjögren patients. The bracket indicates the statistical comparison with the corresponding *p* value. Red horizontal lines indicate the cutoff for increased radiosensitivity at ≥0.55 breaks per metaphase; dashed black horizontal lines are set at 0.5 B/M. Purple upward triangles indicate non-oncological Sjögren patients and pink downward triangles indicate oncological Sjögren patients; dashed blue lines indicate linear regression lines. ANA = antinuclear antibody; B/M = breaks per metaphase. Statistical analyses were performed using simple linear regression for (**B**,**E**) and an unpaired *t*-test with Welch’s correction for (**F**).

ANA titers showed a heterogeneous distribution in both groups, with non-radiosensitive patients predominantly exhibiting lower titers, whereas radiosensitive patients demonstrated a relative shift toward higher titers (Figure 5A). This pattern was also reflected by a weak positive trend in regression analysis, but did not reach statistical significance (R^2^ = 0.051, *p* = 0.222) (Figure 5B). ANA staining patterns were broadly comparable across groups, with a clear predominance of the granular pattern throughout the cohort (Figure 5C). Beyond ANA characteristics, the overall number of detectable autoantibodies per patient was assessed as a surrogate marker of systemic autoimmune burden. The distribution of antibody counts suggested a modest shift toward higher values in radiosensitive individuals (Figure 5D). However, regression analysis revealed only a weak, non-significant association (R^2^ = 0.027, *p* = 0.378) between antibody number and radiosensitivity (Figure 5E). Consistently, comparison of mean antibody counts between groups confirmed a slight but non-significant increase in radiosensitive patients (*p* = 0.546) (Figure 5F).

**Figure 6:**
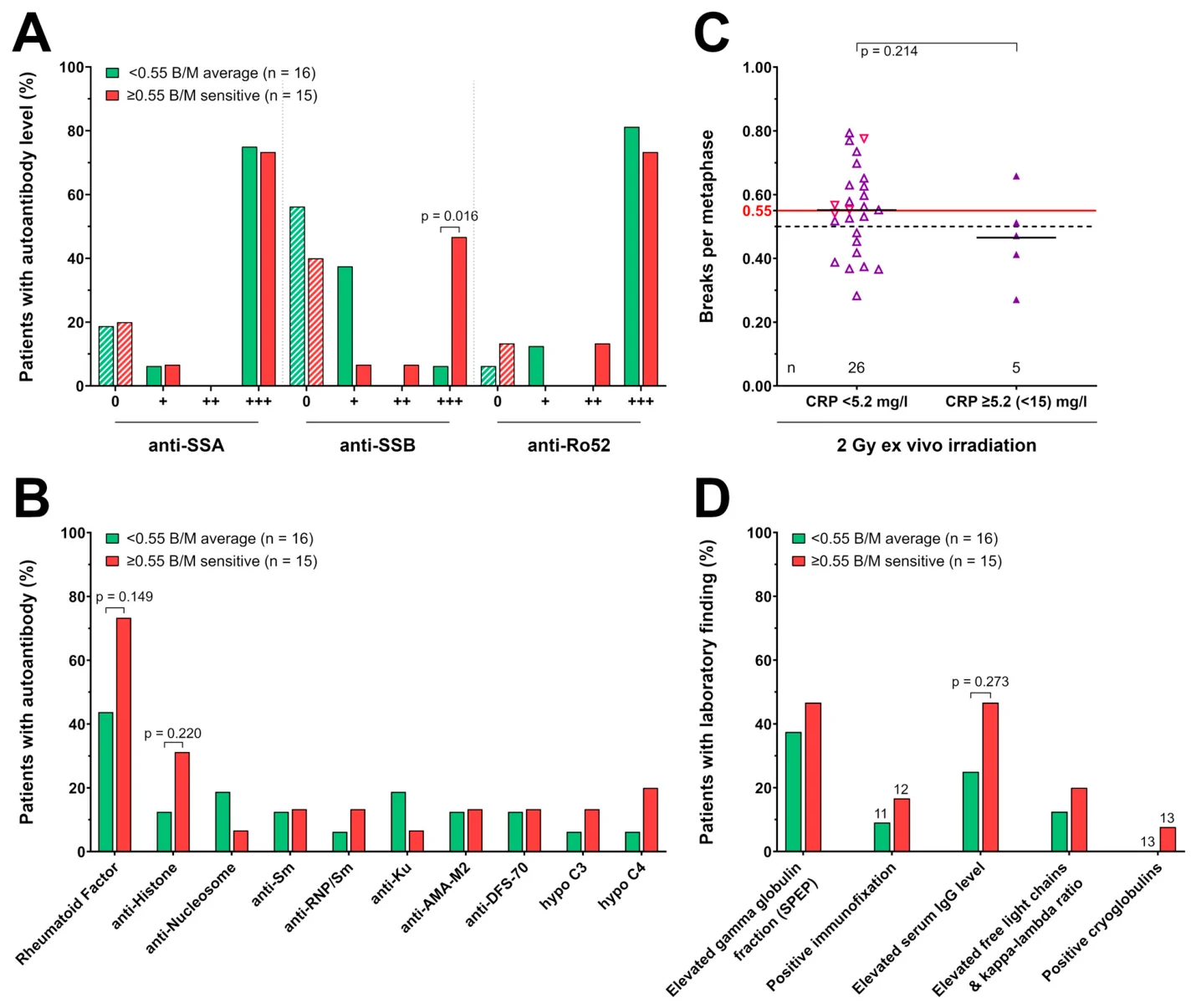
Association of specific autoantibodies and additional laboratory findings with radiosensitivity in the deeply phenotyped Sjögren cohort. (**A**) Distribution of specific Sjögren-associated autoantibodies in non-radiosensitive <0.55 B/M (green bars) and radiosensitive ≥0.55 B/M (red bars) Sjögren patients. (**B**) Distribution of additional autoantibodies and abnormalities in complement levels in non-radiosensitive and radiosensitive Sjögren patients. (**C**) Background-corrected breaks per metaphase after 2 Gy irradiation stratified by CRP levels. (**D**) Distribution of additional laboratory abnormalities in non-radiosensitive and radiosensitive Sjögren patients. Numbers above immunofixation and cryoglobulin bars indicate the respective subgroup sizes due to incomplete data availability. Black horizontal lines indicate mean values. Brackets indicate statistical comparisons with corresponding *p* values. The red horizontal line indicates the cutoff for increased radiosensitivity at ≥0.55 breaks per metaphase; the dashed black horizontal line is set at 0.5 B/M. Purple upward triangles indicate non-oncological Sjögren patients and pink downward triangles indicate oncological Sjögren patients. B/M = breaks per metaphase; CRP = C-reactive protein; SPEP = serum protein electrophoresis; IgG = immunoglobulin G. Statistical analyses were performed using Fisher’s exact tests for (**A**,**B**,**D**) and the Mann–Whitney U test for (**C**).

To refine this analysis, specific Sjögren-associated autoantibodies were examined (Figure 6A). Anti-SSA and anti-Ro52 antibodies showed largely comparable distributions between radiosensitive and non-radiosensitive individuals, with most patients exhibiting high-grade (+++) expression in both groups. In contrast, anti-SSB antibodies demonstrated a distinct pattern. High-grade anti-SSB positivity was observed in 7 of 15 radiosensitive patients, compared with 1 of 16 non-radiosensitive patients, corresponding to an odds ratio of 13.13 (95% CI 1.52–155.6). This difference was statistically significant in the unadjusted analysis (Fisher’s exact *p* = 0.016), but did not remain statistically significant after Benjamini–Hochberg correction for multiple testing (*q* = 0.124). Nevertheless, high-grade anti-SSB positivity emerged as the strongest serological correlate of radiosensitivity in this exploratory analysis, highlighting a potentially relevant association.

Other autoantibodies and abnormalities in serum complement C3 and C4 levels were largely similarly distributed between groups (Figure 6B). More pronounced differences were observed for anti-histone antibodies and rheumatoid factor, which were more frequent in radiosensitive patients, but did not reach statistical significance (rheumatoid factor: *p* = 0.149; anti-histone: *p* = 0.220). Likewise, CRP-based stratification did not reveal a relevant association (*p* = 0.214) between systemic inflammatory status and radiosensitivity (Figure 6C). However, interpretation was limited by overall low CRP levels and markedly imbalanced group sizes. Additional laboratory abnormalities showed slightly higher frequencies in radiosensitive patients across several parameters (Figure 6D). Overall differences were small, although the most pronounced group difference was observed for elevated IgG levels, albeit without statistical significance (*p* = 0.273).

Altogether, these findings do not support a generalized association between humoral autoimmune activity and radiosensitivity in Sjögren’s syndrome. Rather, the data may point toward a more antibody-specific pattern, with anti-SSB antibody levels showing the most distinct distribution between radiosensitive and non-radiosensitive patients in our cohort. However, given that the association observed for high-grade anti-SSB positivity did not withstand correction for multiple testing, it should be regarded as hypothesis-generating rather than confirmatory. Detailed distributions of laboratory parameters are provided in Supplementary Table S2.

### 3.7. Influence of Immunosuppressive Therapy on Radiosensitivity in Sjögren’s Syndrome

**Figure 7:**
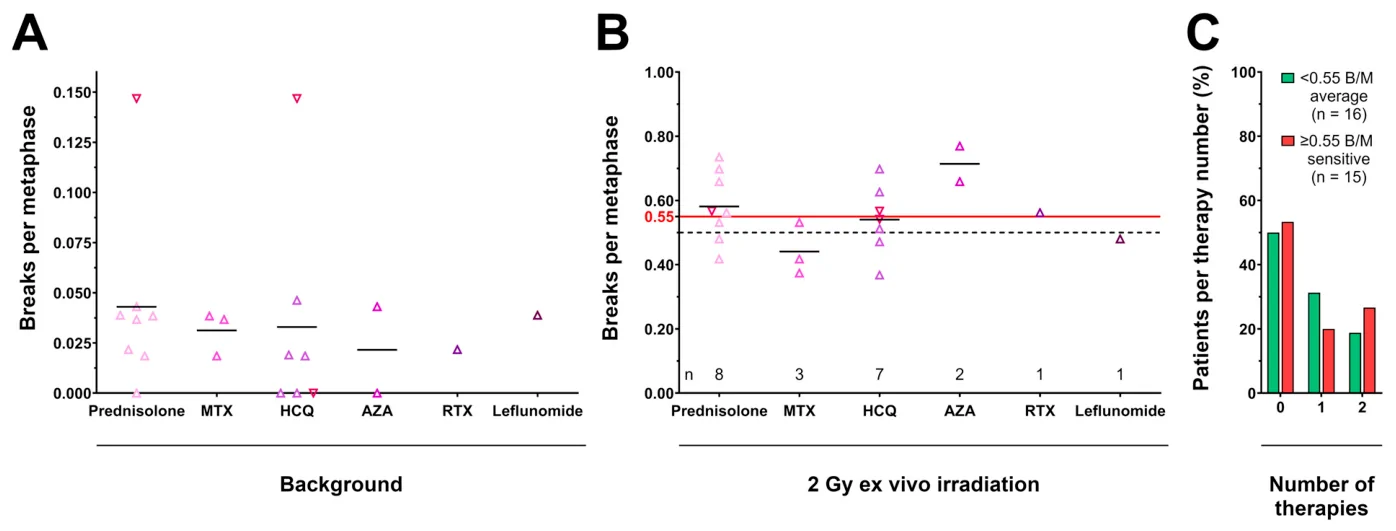
Association of systemic immunosuppressive therapy with chromosomal aberrations and radiosensitivity in the deeply phenotyped Sjögren cohort. (**A**) Background values stratified by immunosuppressive treatment group. (**B**) Background-corrected breaks per metaphase after 2 Gy irradiation according to the same treatment groups. Patients without immunosuppressive therapy are not shown in (**A**,**B**); patients receiving prednisolone in combination with another immunosuppressive agent are included in both corresponding treatment groups. (**C**) Distribution of the number of systemic immunosuppressive therapies in non-radiosensitive (green bars) and radiosensitive (red bars) Sjögren patients. Black horizontal lines indicate mean values. The red horizontal line indicates the cutoff for increased radiosensitivity at ≥0.55 breaks per metaphase; the dashed black horizontal line is set at 0.5 B/M. Upward triangles indicate non-oncological Sjögren patients and pink downward triangles indicate oncological Sjögren patients. MTX = methotrexate; HCQ = hydroxychloroquine; AZA = azathioprine; RTX = rituximab; B/M = breaks per metaphase. Statistical analyses were performed using Kruskal–Wallis tests for (**A**,**B**).

Given that drug effects have been suggested, a final analysis assessed whether immunosuppressive therapy has a potential impact on chromosomal instability and radiosensitivity in patients with Sjögren’s syndrome. The analysis included all immunosuppressive agents present in the deeply phenotyped subcohort, namely prednisolone, methotrexate (MTX), hydroxychloroquine (HCQ), azathioprine, rituximab, and leflunomide. Neither baseline chromosomal aberrations (Figure 7A) nor radiosensitivity after 2 Gy irradiation (Figure 7B) differed significantly between treatment groups (Background: *p* = 0.566; 2 Gy: *p* = 0.183), as assessed using the Kruskal–Wallis test in groups with sufficient sample size (prednisolone, MTX, HCQ). An apparent increase in mean radiosensitivity in the azathioprine subgroup could not be evaluated statistically due to limited sample size. Consistently, the cumulative number of immunosuppressive agents per patient (0, 1, or 2 drugs) was largely comparable between radiosensitive and non-radiosensitive individuals, indicating no relevant association with radiosensitivity (Figure 7C). Detailed distributions of therapies across subgroups are provided in Supplementary Table S3.

## 4. Discussion

In this study, we investigated ex vivo chromosomal radiosensitivity in patients with Sjögren’s syndrome using a three-color FISH assay and compared non-oncological and oncological Sjögren patients with healthy controls, breast cancer patients, and previously characterized SLE cohorts. We found that patients with Sjögren’s syndrome showed significantly increased radiosensitivity after 2 Gy ex vivo irradiation compared with healthy controls. Notably, this increase was already present in the non-oncological Sjögren cohort, while individuals with concomitant malignancy had no further increase in radiosensitivity. This contrasts with the previously characterized SLE cohort from our institution, in which non-oncological SLE patients were comparable to healthy controls, whereas increased mean radiosensitivity was mainly observed in patients with cancer. When searching for factors associated with increased radiosensitivity within the deeply phenotyped Sjögren cohort, high-grade anti-SSB positivity emerged as the strongest serological signal in the exploratory analysis, whereas no clear association was observed with general demographic, disease-related, clinical, laboratory, or treatment-related parameters.

Regarding Sjögren’s syndrome, a literature search reveals that available evidence on radiotherapy-related toxicity is limited and largely restricted to individual case reports. One early case report published in 1996 described severe mucosal and soft tissue toxicity following chemoradiotherapy for nasopharyngeal carcinoma in a patient with Sjögren’s syndrome, including extensive ulceration and retropharyngeal abscess formation exceeding expected radiation-induced effects. The authors emphasized the need for caution when applying combined modality treatment in patients with underlying connective tissue disease [9]. Similarly, a recent case report published in 2025 reported pronounced acute and late toxicities during definitive chemoradiotherapy for HPV-positive oropharyngeal carcinoma in a 43-year-old Japanese woman with Sjögren’s syndrome, including severe radiation dermatitis, confluent mucositis, and persistent xerostomia [10]. Although these observations suggest a potentially increased susceptibility to radiotherapy-related toxicity in Sjögren’s syndrome, the currently available evidence remains insufficient to draw robust conclusions regarding the magnitude of risk and requires further systematic investigation. However, the hypothesis that connective tissue diseases (CTDs) may be associated with increased susceptibility to ionizing radiation and an elevated risk of radiotherapy-related toxicity has been discussed for several decades and is supported by a broader body of clinical and experimental evidence. This concept has been investigated both clinically, through studies assessing radiotherapy-associated toxicity, and experimentally, through laboratory studies examining cellular radiosensitivity. Several clinical studies support an association between CTDs and increased risk of radiotherapy-related toxicity. A large retrospective cohort study evaluated radiotherapy-associated complications in 209 patients with various CTDs, among them 131 patients with rheumatoid arthritis (RA) (Rheumatoid arthritis is usually classified separately from systemic connective tissue diseases in the narrower rheumatological sense. However, several studies in the radiotherapy literature include rheumatoid arthritis in broader CTD cohorts), 25 with systemic lupus erythematosus (SLE), and 16 with scleroderma/systemic sclerosis (SSc). Severe late radiation effects occurred significantly more frequently in non-RA CTDs, whereas RA itself did not appear to be associated with an increased rate of late toxicity. These findings suggest that the risk of radiotherapy-associated complications may differ between CTD subtypes, with potentially higher susceptibility in non-RA disease entities [11]. Similar findings were reported in a matched case–control study examining 73 patients with several CTDs, including 33 patients with RA, 13 with SLE, and 9 with SSc. This study revealed significantly increased late radiotherapy-related toxicity in patients with CTDs overall. The increase in toxicity was most pronounced in patients with SLE and SSc. In contrast, no significant differences between CTD patients and matched controls were observed regarding acute toxicities [12]. These observations were further supported by a recent meta-analysis published in 2021, including 10 controlled studies comprising 4028 patients (406 with CTDs and 3622 controls). The analysis demonstrated a statistically significant increase in both acute and late moderate-to-severe radiotherapy-related toxicities in patients with CTDs compared with controls, although severe toxicities remained uncommon overall. Subgroup analyses identified increased toxicity in patients with SLE and SSc, but also in patients with RA [13]. However, not all studies have confirmed an increased risk of radiotherapy-associated toxicity in patients with CTDs. An earlier matched case–control study comprising 38 patients, including 21 patients with SLE and smaller numbers of other CTDs, found no significant differences in either acute or late toxicity compared with matched controls. Notably, this study was the only one among the above-mentioned investigations to include a small subgroup of patients with Sjögren’s syndrome. However, no increased toxicity was observed in these patients, although the very small patient number substantially limited interpretability. The only CTD subgroup showing a slight increase in acute and late complications consisted of two patients with SSc. Overall, these findings contrast with the previously described studies and highlight the heterogeneity of the currently available clinical data regarding radiotherapy-associated toxicity in CTDs [23]. Despite this heterogeneity, systemic sclerosis and systemic lupus erythematosus emerge as the CTD subtypes most consistently associated with increased radiotherapy-related toxicity across the previously described studies and have also been investigated in disease-specific retrospective cohort studies. These studies likewise demonstrated acute and chronic radiotherapy-related toxicities in a substantial proportion of patients with SSc and SLE, further supporting the concept that susceptibility to radiation-induced tissue injury may differ between CTD subtypes, with certain disease entities showing more consistent signals than others. In SLE, toxicity correlated with specific clinical features of disease severity, suggesting that more advanced or systemic disease manifestations may confer higher susceptibility to radiation-induced damage. Nevertheless, radiotherapy was not considered an absolute contraindication in either SSc or SLE cohorts, but rather a therapeutic option requiring careful individual risk–benefit assessment [24,25,26]. SLE and SSc, the CTD entities most consistently associated with radiotherapy-related toxicity in clinical studies, have also shown experimental evidence suggestive of increased cellular radiosensitivity in lymphocyte-based assays assessing radiation-induced DNA damage [27]. However, findings from other lymphocyte-based experimental assays are not fully consistent. A case–control laboratory study in SLE found no overall increase in intrinsic cellular radiosensitivity compared with healthy controls, although higher levels of radiation-induced DNA damage were associated with specific clinical and serological features [28]. Another study reported increased radiosensitivity in clinically active SLE, with normalization during remission, suggesting a potential influence of disease activity on cellular radiation response [29]. FISH-based studies from our institution further support this heterogeneous picture. In a cohort of 70 patients with SLE, mean radiosensitivity was comparable to healthy controls in the non-oncological cohort but significantly increased in patients with concomitant malignancy. Nevertheless, compared with healthy controls, both SLE cohorts showed a higher proportion of individuals exceeding the radiosensitivity threshold of ≥0.5 breaks per metaphase, including the non-oncological SLE cohort despite its comparable mean radiosensitivity [14]. These findings indicate marked interindividual variability, with radiosensitive individuals present even in cohorts without increased mean radiosensitivity. Similarly, a further study from our institution, which focused primarily on rheumatoid arthritis but also included other rheumatic and connective tissue diseases, found no increased mean radiosensitivity in RA patients with or without malignancy, although individual radiosensitive patients were identified, particularly among oncological RA patients [30]. Of note, the small Sjögren subgroup included in this previous study did not show increased mean radiosensitivity, but its size was too limited to allow reliable conclusions for Sjögren’s syndrome specifically. Taken together, available clinical and experimental data suggest that radiation susceptibility in CTDs is heterogeneous, with both disease-specific patterns and marked interindividual variability within individual disease entities.

To better understand why patients with systemic autoimmune diseases might be more susceptible to ionizing radiation, it is useful to consider radiosensitivity from a mechanistic perspective. The initial amount of radiation-induced DNA damage is thought to vary only moderately between individuals, whereas relevant interindividual differences arise mainly during the subsequent cellular response to this damage, including DNA damage recognition, repair pathway activation, cell-cycle control, and cell fate decisions [16]. Chronic systemic autoimmune diseases are characterized by persistent inflammation, oxidative stress, accumulation of endogenous DNA damage, and epigenetically regulated functional abnormalities in DNA damage response and repair pathways [31]. This has also been described in CTDs such as SLE, where increased DNA damage and impaired repair mechanisms have been implicated in disease pathogenesis [32]. By involving biological processes that are central to radiation response, pre-existing disturbances in DNA damage response and repair may therefore reduce the capacity to cope with additional radiation-induced DNA damage.

In our study, anti-SSB positivity showed the strongest unadjusted association with increased radiosensitivity. Although this finding did not remain statistically significant after correction for multiple testing, it raises the question of whether anti-SSB positivity represents an incidental finding or a specific signal linked to altered radiation response. From a cellular perspective, SSB/La is an RNA-binding protein involved in RNA metabolism, particularly in the synthesis and maturation of RNA polymerase III transcripts [33]. Beyond this function, SSB/La has been shown to participate in oxidative stress responses by translocating from the nucleus to the cytoplasm, binding to the 5′UTR of Nrf2 mRNA and facilitating stress-induced translation of Nrf2, a key regulator of antioxidant defense [34]. In the context of ionizing radiation, where oxidative stress and DNA damage are central biological events [35], anti-SSB-associated interference with SSB/La-related stress-response pathways could therefore present one possible explanation for increased radiosensitivity. Another hypothesis arises from a peptide-based epitope mapping study showing 72% sequence similarity between a SSB/La epitope and a fragment of human DNA topoisomerase II; anti-SSB/La antibodies showed cross-reactivity with peptides derived from these homologous regions [36]. Given the role of topoisomerase II in DNA topology and genomic stability, such cross-reactivity could theoretically affect DNA integrity and thereby increase susceptibility to DNA damage in anti-SSB-positive patients. However, these mechanisms remain speculative, and current data do not demonstrate that anti-SSB antibodies have functional consequences for oxidative stress defense, DNA repair, chromosomal stability or radiation response. Overall, the association between anti-SSB positivity and increased radiosensitivity in our cohort should be regarded as an exploratory but biologically plausible signal that requires validation in larger cohorts and functional studies addressing oxidative stress defense, DNA damage response, and radiation sensitivity.

In contrast to the SLE cohort from our institution, in which increased mean radiosensitivity was mainly observed in patients with concomitant malignancy [14], our Sjögren cohort showed elevated radiosensitivity already in the non-oncological subgroup, with comparable values in oncological and non-oncological patients. A contribution of malignancy to radiosensitivity is biologically plausible, as cancer is associated with genomic instability, increased DNA damage, DNA repair defects, and impaired cell-cycle control [37]. Accordingly, FISH-based studies have shown increased chromosomal radiosensitivity in different cancer cohorts compared with healthy controls [38,39]. In patients with both SLE and cancer, cancer-associated DNA damage and autoimmune disease-related alterations in DNA damage response may therefore act additively or synergistically. By comparison, the increased radiosensitivity already observed in our non-oncological Sjögren cohort may indicate a stronger contribution of disease-associated factors less dependent on concomitant malignancy. However, given the limited cohort sizes and the unmatched observational design, an additional influence of malignancy cannot be excluded. In general, interpretation of oncological cohorts remains challenging, as previous or concomitant anticancer therapies may additionally influence DNA damage accumulation and repair capacity, given that both chemotherapy and radiotherapy exert major effects through DNA damage and DNA damage response pathways [40,41]. Thus, treatment-related confounding cannot be fully excluded. Another notable difference between the two studies concerns the associated autoantibody pattern. While the SLE study reported an association between increased radiosensitivity and anti-Ro52 antibodies [14], the most distinct serological pattern in our Sjögren cohort was observed for anti-SSB positivity. These findings may reflect disease-specific differences in autoimmune biology, as Ro52 and SSB/La have distinct cellular functions. However, the SSA/Ro and SSB/La antigen systems may not be completely independent. Molecular studies suggest that the 52-kD (Ro52) and 60-kD SSA/Ro components may associate within the cell and that SSA/Ro and SSB/La antigens may occur in complexes during certain stages of the cell [33]. Moreover, although anti-SSB/La was not reported as an associated parameter in the institutional SLE study, anti-SSB/La positivity has previously been associated with increased radiation-induced DNA double-strand breaks in an independent SLE laboratory study [28]. Overall, SLE and Sjögren’s syndrome both belong to the spectrum of CTDs and share autoimmune and pathogenetic features such as chronic inflammation, B-cell activation, autoantibody production and interferon-driven immune activation, while differing in clinical, serological and organ-specific manifestations. Nevertheless, their underlying pathogenic mechanisms remain only partially understood and are considered highly multifactorial [15]. Importantly, both the SLE and Sjögren studies showed pronounced interindividual variability in radiosensitivity within their respective patient cohorts. This intra-disease heterogeneity argues against a single uniform disease-specific mechanism of radiosensitivity and rather suggests a complex interplay of multiple, only partially defined and potentially disease-independent mechanisms. The existence of CTD overlap syndromes further highlights the complexity of the underlying disease biology and makes the attribution of radiosensitivity patterns to specific disease mechanisms or molecular pathways even more challenging.

Sjögren’s syndrome has been associated with increased all-cause mortality, with a meta-analysis including 14 studies and 14,584 patients reporting an approximately 50% higher mortality risk compared with the general population [4]. Leading causes of death include respiratory and circulatory diseases, as well as malignancies [42]. The increased cancer risk in Sjögren’s syndrome involves selected solid tumors, such as thyroid cancer, but is most consistently described for hematological malignancies, particularly non-Hodgkin lymphoma [5,43]. Radiotherapy remains a central component of oncological treatment, with approximately half of all cancer patients receiving radiotherapy at some point during their disease course [6]. It is also clinically relevant in non-Hodgkin lymphoma, both in curative and consolidative treatment settings [7]. Despite technical advances, radiotherapy can still cause substantial acute and late toxicities, including fatigue, mucosal reactions, xerostomia, fibrosis and organ-specific complications [8]. In rare cases, severe toxicity may be fatal, as illustrated by a case report of lethal late toxicity after prostate cancer radiotherapy in a patient with 35–55% increased FISH-based radiosensitivity [44]. At the same time, the clinical manifestation of radiation toxicity depends not only on individual radiosensitivity but also on treatment-related factors such as radiation dose, fractionation, irradiated volume, and treatment site. Nevertheless, several studies suggest that laboratory radiosensitivity assays, including approaches evaluating DNA repair capacity, residual DNA damage, or chromosomal radiosensitivity, may reflect an individual predisposition to radiation-induced normal-tissue toxicity [16,45,46]. In our previously characterized SLE cohort, radiosensitivity testing was used to guide dose adaptation in selected oncological patients, demonstrating that assay-based treatment adaptation can be clinically implemented in a related connective tissue disease. Dose reduction was recommended in 11 patients and implemented in five, with no locoregional relapse observed during the reported follow-up [14]. However, the present study did not assess radiotherapy-related toxicity or treatment adaptation in patients with Sjögren’s syndrome and therefore does not allow specific clinical treatment recommendations to be derived. Prospective studies directly correlating ex vivo radiosensitivity with clinical radiotherapy toxicity and treatment outcomes are required to establish the clinical utility of this approach in Sjögren’s syndrome. In this context, the potential future clinical relevance of radiosensitivity assays may lie in the identification of patients with increased susceptibility to radiation-induced toxicity, thereby helping to improve the safety of radiotherapy, reduce adverse effects, and preserve radiotherapy as an effective option whenever it can be applied safely. Ultimately, this aligns with a broader paradigm shift in oncology, moving from standardized treatment concepts toward more individualized approaches—a development that is increasingly extending to radiotherapy [47].

The main limitations of this study are the limited cohort size and small subgroup sizes, reducing statistical power, particularly for analyses requiring further stratification, and potentially limiting the detection of smaller associations. In addition, the strong female predominance, although consistent with the epidemiology of Sjögren’s syndrome, limited the assessment of sex-specific effects. Comprehensive clinical, laboratory, and treatment-related data were available only for the deeply phenotyped subcohort, comprising all 27 non-oncological Sjögren patients and the 4 prospectively recruited oncological Sjögren patients. Analyses of factors associated with radiosensitivity were thus limited to this subgroup and should be regarded as exploratory. Potential confounding by malignancy, previous oncological therapies, immunosuppressive treatment, or other clinical variables cannot be fully excluded. Accordingly, the association between high-grade anti-SSB positivity and increased radiosensitivity should be regarded as hypothesis-generating and requires validation in larger independent cohorts. A further clinically relevant question is whether biomarkers exist that are associated both with concomitant malignancy and with increased individual radiosensitivity in Sjögren’s syndrome. Clinical and laboratory features associated with malignancy in Sjögren’s syndrome have predominantly been described in the context of lymphoma development, including persistent salivary gland enlargement, lymphadenopathy, cryoglobulinemia, low complement levels, and monoclonal gammopathy [48,49]. However, their potential relationship to individual radiosensitivity remains unknown. Although several of these parameters were included in our exploratory analyses, no significant associations with radiosensitivity were identified, while others were too infrequent or incompletely available for meaningful evaluation. Accordingly, the present study was limited in its ability to reliably assess potential biomarkers associated with both malignancy and radiosensitivity, particularly given the small number of comprehensively phenotyped oncological patients (*n* = 4), only two of whom had lymphoma. Furthermore, radiotherapy-related toxicity cannot be explained solely by increased ex vivo chromosomal radiosensitivity, as it is also influenced by multiple patient- and treatment-related factors. Finally, the FISH assay depends on successful lymphocyte stimulation. Patients receiving high-dose prednisolone (>20 mg/d) cannot be studied with our assay, because lymphocytes can no longer be reliably stimulated to enter the cell cycle, preventing adequate metaphase generation. Since such patients often have higher disease activity, highly active disease states may be underrepresented.

## 5. Conclusions

In conclusion, patients with Sjögren’s syndrome showed increased ex vivo chromosomal radiosensitivity, already detectable in the absence of malignancy. In contrast to previously characterized SLE cohorts, concomitant cancer did not appear to confer a further increase in radiosensitivity in Sjögren patients, a pattern that may indicate a stronger contribution of disease-associated factors, while an additional influence of malignancy cannot be excluded. High-grade anti-SSB positivity showed the strongest unadjusted association with increased radiosensitivity; however, this finding did not remain statistically significant after correction for multiple testing and should therefore be regarded as hypothesis-generating, requiring validation in larger independent cohorts. The translation of these ex vivo findings into clinical practice in Sjögren’s syndrome requires further evaluation. Prospective studies in Sjögren patients undergoing radiotherapy should therefore integrate ex vivo chromosomal radiosensitivity with systematic assessment of radiotherapy-related toxicity and treatment outcomes to further define its role in individualized risk assessment and radiotherapy planning in this patient population.

## Figures and Tables

**Table 1:** Age- and sex-adjusted comparisons of background-corrected breaks per metaphase after 2 Gy irradiation.

Adjusted Comparison	Adjusted β (B/M)	[95% CI] (B/M)	*p* Value
Breast cancer vs. Healthy	0.036	[0.007 to 0.066]	<0.001
SLE non-onco vs. Healthy	−0.011	[−0.053 to 0.031]	0.611
Sjögren non-onco vs. Healthy	0.120	[0.071 to 0.168]	<0.001
SLE onco vs. Breast cancer	0.068	[0.022 to 0.114]	0.004
Sjögren onco vs. Breast cancer	0.080	[0.021 to 0.139]	0.008
Sjögren non-onco vs. SLE non-onco	0.127	[0.059 to 0.195]	<0.001
Sjögren onco vs. SLE onco	0.012	[−0.057 to 0.081]	0.734
SLE onco vs. SLE non-onco	0.115	[0.059 to 0.171]	<0.001
Sjögren onco vs. Sjögren non-onco	−0.003	[−0.075 to 0.068]	0.924

Adjusted β represents the estimated between-group difference in background-corrected B/M after adjustment for age and sex; positive values indicate higher B/M in the first-mentioned cohort.

**Table 2:** Cancer types in the oncological Sjögren’s syndrome cohort stratified by radiosensitivity.

Cancer Types	Frequency of Cancer Type, *n* (%)		
	Sjögren Onco		
		Radiosensitivity	
	All	Normal	Increased
	*n* = 18	*n* = 8	*n* = 10
**MALT lymphoma**	4 (22.2%)	3 (37.5%)	1 (10%)
Salivary glands (parotid, submandibular gland)	2 (11.1%)	1 (12.5%)	1 (10%)
Palate	1 (5.6%)	1 (12.5%)	0 (0%)
Cervical lymph node	1 (5.6%)	1 (12.5%)	0 (0%)
**Head and neck tumors (HNTs)**	2 (11.1%)	1 (12.5%)	1 (10%)
Parotid carcinoma	1 (5.6%)	1 (12.5%)	0 (0%)
Lateral tongue carcinoma	1 (5.6%)	0 (0%)	1 (10%)
**Gastrointestinal tumors (GITs)**	3 (16.7%)	1 (12.5%)	2 (20%)
Esophageal squamous cell carcinoma	1 (5.6%)	0 (0%)	1 (10%)
Anal carcinoma	1 (5.6%)	1 (12.5%)	0 (0%)
Pancreatic carcinoma	1 (5.6%)	0 (0%)	1 (10%)
**Breast carcinoma**	7 (38.9%)	3 (37.5%)	4 (40%)
**Other gynecological tumors**	1 (5.6%)	0 (0%)	1 (10%)
Serous adenocarcinoma of the ovary/fallopian tube/peritoneum	1 (5.6%)	0 (0%)	1 (10%)
**Lung carcinoma**	1 (5.6%)	0 (0%)	1 (10%)

The Bold text indicates the main cancer categories, while non bold entries specify the corresponding subtypes.

## Data Availability

The datasets generated and/or analyzed in the present study are not publicly accessible due to privacy protection policy, but can be provided by the corresponding author upon reasonable request.

## Supplementary Materials

The following supporting information can be downloaded at https://www.mdpi.com/article/10.3390/cells15201855/s1. Table S1: Baseline characteristics and clinical features of non-oncological and oncological Sjögren’s syndrome patients; Table S2: Diagnostic and laboratory characteristics of non-oncological and oncological Sjögren’s syndrome patients stratified by radiosensitivity; Table S3: Systemic immunosuppressive therapy in non-oncological and oncological Sjögren’s syndrome patients stratified by radiosensitivity; Table S4: Descriptive statistics and effect estimates for unadjusted primary cohort comparisons of background chromosomal aberrations and background-corrected breaks per metaphase after 2 Gy irradiation.

## Abbreviations

The following abbreviations are used in this manuscript:

CTDs	connective tissue diseases
ESSDAI	EULAR Sjögren’s Syndrome Disease Activity Index
FISH	fluorescence in situ hybridization
RA	rheumatoid arthritis
SLE	systemic lupus erythematosus
SSc	scleroderma/systemic sclerosis
