# Supplementary material for: Chromosomal Radiosensitivity in Sjögren’s Syndrome with and Without Concomitant Malignancy
Source: Cells. 2026 Oct 13;15(20):1855. doi: 10.3390/cells15201855 (PMC13703102; doi:10.3390/cells15201855)
Supplement: Supplementary file 1 [file cells-15-01855-s001.zip › cells-4552488-supplementary.pdf]

Supplementary Table S1. Baseline characteristics and clinical features of non-oncological and oncological Sjögren's syndrome patients.

			Baseline characteristics and clinical features (%) [no data]	
			**Sjögren non-onco**	**Sjögren onco**
			n = 27	n = 18
		**Sex (female/male)**	25/2 (92.6%/7.4%)	18/0 (100%/0%)
		**Mean age in years (range)**	48.1 (19-74)	58.6 (24-78)
		**Mean age at diagnosis of Sjögren in years (range)**	41.7 (17-72)	44.5 (15-69) [14]
		**Mean time (years) between Sjögren diagnosis and blood sampling for laboratory analysis (range)**	6.4 (0-19)	8.3 (1-15) [14]
**Body measure-ments**		**Mean height in cm (standard deviation)**	165.6 (7.1)	168.8 (7.2) [14]
		**Mean weight in kg (standard deviation)**	65.8 (15.4)	71 (5.8) [14]
		**Mean BMI (standard deviation)**	24.1 (6.1)	25.1(3.6) [14]
	**BMI**	15 ≤ BMI < 20	7 (25.9%)	0(0%)
		20 ≤ BMI < 25	11 (40.7%)	3(75%)
		25 ≤ BMI < 30	5 (18.5%)	0(0%)
		30 ≤ BMI	4 (14.8%)	1(25%)
**Rheumatologic comorbidities**		Systemic Lupus Erythematosus (SLE)	2 (7.4%)	4 (57.1%) [11]
		Rheumatoid arthritis (RA)	1 (3.7%)	1 (14.3%) [11]
		Dermatomyositis	1 (3.7%)	0 (0%) [11]
**Other frequently observed comor-bidities**		Arterial hypertension (aHT)	6 (22.2%)	2 (50%) [14]
		Obesity	4 (14.8%)	1 (25%) [14]
		Hypothyroidism	6 (22.2%)	1 (25%) [14]
		Hashimoto's thyroiditis	7 (25.9%)	0 (0%) [14]
		Thyroid nodules / nodular goiter	4 (14.8%)	1 (25%) [14]
		Bronchial asthma	4 (14.8%)	0 (0%) [14]
		Finger polyarthrosis	2 (7.4%)	1 (25%) [14]
		Chronic pain syndrome	4 (14.8%)	0 (0%) [14]
		Depression	3 (11.1%)	0 (0%) [14]
**ESSDAI-relevant symptoms**		Fever / weight loss	1 (3.7%)	0 (0%) [14]
		Lymphadenopathy / lymphoma	3 (11.1%)	2 (50%) [14]
		Glandular swelling (salivary / lacrimal glands)	12 (44.4%)	4 (100%) [14]
		Skin involvement (erythema multiforme / limited or diffuse cutaneous vasculitis)	0 (0%)	0 (0%) [14]
		Pulmonary involvement	2 (7.4%)	0 (0%) [14]
		Renal involvement	0 (0%)	0 (0%) [14]
		Myopathy / myositis	0 (0%)	0 (0%) [14]
		Hematologic involvement (cytopenias)	5 (18.5%)	0 (0%) [14]
		Biological / serological abnormalities (complement / immuno- / cryogloblins)	10 (37%)	3 (75%) [14]
		complement	0 (0%)	1 (25%)
		immunoglobulins	6 (22.2%)	2 (50%)
		complement + immunoglobulins	3 (11.1%)	0 (0%)
		complement + cryoglobulins	1 (3.7%)	0 (0%)
		Central nervous system involvement	1 (3.7%)	0 (0%) [14]
**Mean ESSDAI score**			4 (5/0-23)	7.3 (5.5/2-16) [14]

(standard deviation /range)			
**Other Clinical Features**	Sicca symptoms	27 (100%)	5 (100%) [13]
	Raynaud's phenomenon	12 (44.4%)	1 (25%) [14]
	Joint involvement	23 (85.2%)	3 (75%) [14]
	subjective (non-verifiable)	6 (22.2%)	0 (0%)
	objective (clinically verifiable)	17 (63%)	3 (75%) [14]
	Gastrointestinal involvement (esophagus / liver / pancreas)	2 (7.4%)	0 (0%) [14]
	esophagus	1 (3.7%)	---
	liver	1 (3.7%)	---
	pancreas	0 (0%)	---

Missing data are indicated in square brackets. BMI = body mass index; ESSDAI = EULAR Sjögren's Syndrome Disease Activity Index.

Supplementary Table S2. Diagnostic and laboratory characteristics of non-oncological and oncological Sjögren's syndrome patients stratified by radiosensitivity.

		Diagnostic and laboratory characteristics (%) [no data]					
		Sjögren non-onco			Sjögren onco		
			radiosensitivity			radiosensitivity	
	Diagnostics	all	normal	increased	all	normal	increased
		n = 27	n = 15	n = 12	n = 18	n = 8	n = 10
Auto-antibodies	**Presence of auto-antibodies**	27 (100%)	15 (100%)	12 (100%)	4 (100%) [14]	1 (100%) [7]	3 (100%) [7]
	**Mean number of autoantibodies / standard deviation (range)**	4.3/1.8 (1-8)	3.9/1.5 (1-7)	4.8/2 (1-8)	4.8/1.9 (3-8) [14]	8/0 (8-8) [7]	3.7/0.5 (3-4) [7]
	**Specific autoantibodies**						
	**anti-SSA (Ro)**	22 (81.5%)	12 (80%)	10 (83.3%)	3 (75%) [14]	1 (100%) [7]	2 (66.7%) [7]
	0	5 (18.5%)	3 (20%)	2 (16.7%)	1 (25%)	0 (0%)	1 (33.3%)
	+	2 (7.4%)	1 (6.7%)	1 (8.3%)	0 (0%)	0 (0%)	0 (0%)
	++	0 (0%)	0 (0%)	0 (0%)	0 (0%)	0 (0%)	0 (0%)
	+++	20 (74.1%)	11 (73.3%)	9 (75%)	3 (75%)	1 (100%)	2 (66.7%)
	**anti-SSB (La)**	13 (48.1%)	6 (40%)	7 (58.3%)	3 (75%) [14]	1 (100%) [7]	2 (66.7%) [7]
	0	14 (51.9%)	9 (60%)	5 (41.7%)	1 (25%)	0 (0%)	1 (33.3%)
	+	7 (25.9%)	6 (40%)	1 (8.3%)	0 (0%)	0 (0%)	0 (0%)
	++	1 (3.7%)	0 (0%)	1 (8.3%)	0 (0%)	0 (0%)	0 (0%)
	+++	5 (18.5%)	0 (0%)	5 (41.7%)	3 (75%)	1 (100%)	2 (66.7%)
	**anti-Ro52**	24 (88.9%)	14 (93.3%)	10 (83.3%)	4 (100%) [14]	1 (100%) [7]	3 (100%) [7]
	0	3 (11.1%)	1 (6.7%)	2 (16.7%)	0 (0%)	0 (0%)	0 (0%)
	+	2 (7.4%)	2 (13.3%)	0 (0%)	0 (0%)	0 (0%)	0 (0%)
	++	2 (7.4%)	0 (0%)	2 (16.7%)	0 (0%)	0 (0%)	0 (0%)
	+++	20 (74.1%)	12 (80%)	8 (66.7%)	4 (100%)	1 (100%)	3 (100%)
	**Other autoantibodies**	24 (88.9%)	12 (80%)	12 (100%)	4 (100%) [14]	1 (100%) [7]	3 (100%) [7]
	Rheumatoid Factor	14 (51.9%)	6 (40%)	8 (66.7%)	4 (100%)	1 (100%)	3 (100%)
	anti-Histone	3 (11.1%)	1 (6.7%)	2 (16.7%)	1 (25%)	1 (100%)	0 (0%)
	anti-Nucleosome	3 (11.1%)	2 (13.3%)	1 (8.3%)	1 (25%)	1 (100%)	0 (0%)
	anti-Sm	3 (11.1%)	1 (6.7%)	2 (16.7%)	1 (25%)	1 (100%)	0 (0%)
	anti-RNP/Sm	2 (7.4%)	0 (0%)	2 (16.7%)	0 (0%)	0 (0%)	0 (0%)
	anti-Ku	4 (14.8%)	3 (20%)	1 (8.3%)	0 (0%)	0 (0%)	0 (0%)
	anti-AMA-M2	4 (14.8%)	2 (13.3%)	2 (16.7%)	0 (0%)	0 (0%)	0 (0%)
	anti-DFS-70	4 (14.8%)	2 (13.3%)	2 (16.7%)	0 (0%)	0 (0%)	0 (0%)
	**ANA titer (1:x)**						
	negative	1 (3.7%)	0 (0%)	1 (8.3%)	0 (0%) [14]	0 (0%) [7]	0 (0%) [7]
	100	2 (7.4%)	1 (6.7%)	1 (8.3%)	1 (25%) [14]	0 (0%) [7]	1 (33.3%) [7]
	320	9 (33.3%)	7 (46.7%)	2 (16.7%)	0 (0%) [14]	0 (0%) [7]	0 (0%) [7]
	1000	3 (11.1%)	2 (13.3%)	1 (8.3%)	0 (0%) [14]	0 (0%) [7]	0 (0%) [7]
	3200	8 (29.6%)	4 (26.7%)	4 (33.3%)	2 (50%) [14]	0 (0%) [7]	2 (66.7%) [7]
	10000	4 (14.8%)	1 (6.7%)	3 (25%)	1 (25%) [14]	1 (100%) [7]	0 (0%) [7]
	**ANA pattern**						
	granular	21 (77.8%)	12 (80%)	9 (75%)	3 (75%) [14]	1 (100%) [7]	2 (66.7%) [7]
	nucleolar	3 (11.1%)	2 (13.3%)	1 (8.3%)	0 (0%) [14]	0 (0%) [7]	0 (0%) [7]
	homogeneous	1 (3.7%)	1 (6.7%)	0 (0%)	1 (25%) [14]	0 (0%) [7]	1 (33.3%) [7]
	no pattern (negative ANAs)	1 (3.7%)	0 (0%)	1 (8.3%)	0 (0%) [14]	0 (0%) [7]	0 (0%) [7]

**Laboratory findings**	**Complement consumption**						
	C3						
	normal	26 (96.3%)	15 (100%)	11 (91.7%)	4 (100%) [14]	1 (100%) [7]	3 (100%) [7]
	decreased	1 (3.7%)	0 (0%)	1 (8.3%)	0 (0%) [14]	0 (0%) [7]	0 (0%) [7]
	increased	0 (0%)	0 (0%)	0 (0%)	0 (0%) [14]	0 (0%) [7]	0 (0%) [7]
	C4						
	normal	23 (85.2%)	14 (93.3%)	9 (75%)	3 (75%) [14]	1 (100%) [7]	2 (66.7%) [7]
	decreased	4 (14.8%)	1 (6.7%)	3 (25%)	1 (25%) [14]	0 (0%) [7]	1 (33.3%) [7]
	increased	0 (0%)	0 (0%)	0 (0%)	0 (0%) [14]	0 (0%) [7]	0 (0%) [7]
	**CRP (mg/l)**						
	x ≤ 5.2	22 (81.5%)	11 (73.3%)	11 (91.7%)	4 (100%) [14]	1 (100%) [7]	3 (100%) [7]
	5.2 < x ≤ 15	5 (18.5%)	4 (26.7%)	1 (8.3%)	0 (0%) [14]	0 (0%) [7]	0 (0%) [7]
	**Protein electrophoresis**						
	elevated gamma-globulins	11 (40.7%)	5 (33.3%)	6 (50%)	2 (50%) [14]	1 (100%) [7]	1 (33.3%) [7]
	**Immunofixation**						
	negative	16 (59.3%)	9 (60%)	7 (58.3%)	4 (100%) [14]	1 (100%) [7]	3 (100%) [7]
	positive (no M-gradient)	3 (11.1%)	1 (6.7%)	2 (16.7%)	0 (0%) [14]	0 (0%) [7]	0 (0%) [7]
	positive (M-gradient)	0 (0%)	0 (0%)	0 (0%)	0 (0%) [14]	0 (0%) [7]	0 (0%) [7]
	not tested	8 (29.6%)	5 (33.3%)	3 (25%)	0 (0%) [14]	0 (0%) [7]	0 (0%) [7]
	**Immunoglobulins**						
	elevated IgG	9 (33.3%)	3 (20%)	6 (50%)	2 (50%) [14]	1 (100%) [7]	1 (33.3%) [7]
	elevated free light chains & kappa-lambda ratio	3 (11.1%)	1 (6.7%)	2 (16.7%)	2 (50%) [14]	1 (100%) [7]	1 (33.3%) [7]
	**Cryoglobulins**						
	negative	22 (81.5%)	12 (80%)	10 (83.3%)	3 (75%) [14]	1 (100%) [7]	2 (66.7%) [7]
	positive	1 (3.7%)	0 (0%)	1 (8.3%)	0 (0%) [14]	0 (0%) [7]	0 (0%) [7]
	not tested	4 (14.8%)	3 (20%)	1 (8.3%)	1 (25%) [14]	0 (0%) [7]	1 (33.3%) [7]

Missing data are indicated in square brackets. ANA = antinuclear antibody; CRP = C-reactive protein; IgG = immunoglobulin G.

Supplementary Table S3. Systemic immunosuppressive therapy in non-oncological and oncological Sjögren's syndrome patients stratified by radiosensitivity.

	Patients taking drugs (%) [no data]					
		Sjögren non-onco			Sjögren onco	
		radiosensitivity			radiosensitivity	
**Drugs**	all	normal	increased	all	normal	increased
	n = 27	n = 15	n = 12	n = 18	n = 8	n = 10
**Prednisolone**	7 (25.9%)	3 (20%)	4 (33.3%)	1 (25%) [14]	0 (0%) [7]	1 (33.3%) [7]
x ≤ 2.5 mg	3 (11.1%)	1 (6.7%)	2 (16.7%)	1 (25%)	0 (0%)	1 (33.3%)
2.5 mg < x ≤ 5 mg	4 (14.8%)	2 (13.3%)	2 (16.7%)	0 (0%)	0 (0%)	0 (0%)
**Mean prednisolone dose in mg (standard deviation)**	0.93 (1.74)	0.83 (1.75)	1.04 (1.71)	0.63 (1.08) [14]	0 (0) [7]	0.83 (1.18) [7]
**Methotrexate (MTX)**						
current	3 (11.1%)	3 (20%)	0 (0%)	0 (0%) [14]	0 (0%) [7]	0 (0%) [7]
previous	3 (11.1%)	2 (13.3%)	1 (8.3%)	0 (0%) [14]	0 (0%) [7]	0 (0%) [7]
**Hydroxychloroquine (HCQ)**						
current	5 (18.5%)	3 (20%)	2 (16.7%)	2 (50%) [14]	1 (100%) [7]	1 (33.3%) [7]
previous	2 (7.4%)	1 (6.7%)	1 (8.3%)	1 (25%) [14]	0 (0%) [7]	1 (33.3%) [7]
**Azathioprine (AZA)**						
current	2 (7.4%)	0 (0%)	2 (16.7%)	0 (0%) [14]	0 (0%) [7]	0 (0%) [7]
previous	0 (0%)	0 (0%)	0 (0%)	0 (0%) [14]	0 (0%) [7]	0 (0%) [7]
**Rituximab (RTX)**						
current	1 (3.7%)	0 (0%)	1 (8.3%)	0 (0%) [14]	0 (0%) [7]	0 (0%) [7]
previous	0 (0%)	0 (0%)	0 (0%)	0 (0%) [14]	0 (0%) [7]	0 (0%) [7]
**Leflunomide**						
current	1 (3.7%)	1 (6.7%)	0 (0%)	0 (0%) [14]	0 (0%) [7]	0 (0%) [7]
previous	0 (0%)	0 (0%)	0 (0%)	0 (0%) [14]	0 (0%) [7]	0 (0%) [7]
**Number of current drugs per patient**						
0	14 (51.9%)	8 (53.3%)	6 (50%)	2 (50%) [14]	0 (0%) [7]	2 (66.7%) [7]
1	7 (25.9%)	4 (26.7%)	3 (25%)	1 (25%) [14]	1 (100%) [7]	0 (0%) [7]
2	6 (22.2%)	3 (20%)	3 (25%)	1 (25%) [14]	0 (0%) [7]	1 (33.3%) [7]
**Average number of current drugs per patient (standard deviation)**	0.7 (0.8)	0.7 (0.8)	0.8 (0.8)	0.8 (0.8) [14]	1 (0) [7]	0.7 (0.9) [7]

Missing data are indicated in square brackets.

Supplementary Table S4. Descriptive statistics and effect estimates for unadjusted primary cohort comparisons of background chromosomal aberrations and background-corrected breaks per metaphase after 2 Gy irradiation.

**Comparison:** **Cohort 1** vs. **Cohort 2**	**Cohort 1 mean ± SD** (B/M)	**Cohort 2 mean ± SD** (B/M)	**Mean difference: Cohort 2 – Cohort 1 [95% CI]** (B/M)	**p value**
**Background chromosomal aberrations (0 Gy)**				
**Healthy** vs. **Breast cancer**	0.025 ± 0.021	0.100 ± 0.158	0.075 [0.049 to 0.101]	< 0.001
**Healthy** vs. **SLE non-onco**	0.025 ± 0.021	0.032 ± 0.029	0.006 [– 0.001 to 0.014]	0.075
**Healthy** vs. **Sjögren non-onco**	0.025 ± 0.021	0.022 ± 0.018	– 0.003 [– 0.011 to 0.004]	0.386
**Breast cancer** vs. **SLE onco**	0.100 ± 0.158	0.037 ± 0.038	– 0.063 [– 0.092 to – 0.034]	< 0.001
**Breast cancer** vs. **Sjögren onco**	0.100 ± 0.158	0.042 ± 0.040	– 0.058 [– 0.090 to – 0.026]	< 0.001
**SLE non-onco** vs. **SLE onco**	0.032 ± 0.029	0.037 ± 0.038	0.005 [– 0.010 to 0.020]	0.494
**Sjögren non-onco** vs. **Sjögren onco**	0.022 ± 0.018	0.042 ± 0.040	0.020 [– 0.001 to 0.042]	0.066
**Sjögren non-onco** vs. **SLE non-onco**	0.022 ± 0.018	0.032 ± 0.029	0.010 [0.0002 to 0.019]	0.044
**Sjögren onco** vs. **SLE onco**	0.042 ± 0.040	0.037 ± 0.038	– 0.005 [– 0.029 to 0.019]	0.665
**Background-corrected breaks per metaphase after 2 Gy irradiation**				
**Healthy** vs. **Breast cancer**	0.411 ± 0.088	0.449 ± 0.119	0.038 [0.014 to 0.062]	0.002
**Healthy** vs. **SLE non-onco**	0.411 ± 0.088	0.404 ± 0.148	– 0.007 [– 0.058 to 0.044]	0.786
**Healthy** vs. **Sjögren non-onco**	0.411 ± 0.088	0.527 ± 0.139	0.116 [0.059 to 0.173]	< 0.001
**Breast cancer** vs. **SLE onco**	0.449 ± 0.119	0.521 ± 0.146	0.073 [0.017 to 0.129]	0.012
**Breast cancer** vs. **Sjögren onco**	0.449 ± 0.119	0.527 ± 0.178	0.079 [– 0.014 to 0.172]	0.093
**SLE non-onco** vs. **SLE onco**	0.404 ± 0.148	0.521 ± 0.146	0.117 [0.046 to 0.188]	0.002
**Sjögren non-onco** vs. **Sjögren onco**	0.527 ± 0.139	0.527 ± 0.178	0.000 [– 0.104 to 0.105]	0.995
**Sjögren non-onco** vs. **SLE non-onco**	0.527 ± 0.139	0.404 ± 0.148	– 0.123 [– 0.197 to – 0.050]	0.001
**Sjögren onco** vs. **SLE onco**	0.527 ± 0.178	0.521 ± 0.146	– 0.006 [– 0.109 to 0.097]	0.903

All comparisons were performed using unpaired t tests with Welch's correction. B/M = breaks per metaphase; CI = confidence interval; SD = standard deviation; SLE = systemic lupus erythematosus.
